# Identification of cardiovascular health gene variants related to longevity in a Chinese population

**DOI:** 10.18632/aging.103396

**Published:** 2020-09-07

**Authors:** Li Zhang, Chen Bai, Chao Nie, Xiaoquan Zhu, Huiping Yuan, Liang Sun, Qi Zhou, Xiaoling Li, Xuan Xian, Fan Yang, Guofang Pang, Yuan Lv, Xiaolin Ni, Caiyou Hu, Ze Yang

**Affiliations:** 1 The Key Laboratory of Geriatrics, Beijing Institute of Geriatrics, Beijing Hospital, National Center of Gerontology, National Health Commission, Institute of Geriatric Medicine, Chinese Academy of Medical Sciences, Beijing, P.R. China; 2 Graduate School of Chinese Academy of Medical Science and Peking Union Medical College, Beijing, P.R. China; 3 School of Labor and Human Resources, Renmin University, Beijing, P.R. China; 4 BGI-Shenzhen, Shenzhen, Guangdong, P.R. China; 5 iCarbonX Company Limited, Shenzhen, Guangdong, P.R. China; 6 Shanghai Institute of Medical Genetics, Shanghai Children's Hospital, Shanghai Jiao Tong University School of Medicine, Shanghai, P.R. China; 7 Department of Histo-Embryology, Genetics and Developmental Biology, Shanghai Jiao Tong University School of Medicine, Shanghai, P.R. China; 8 Key Laboratory of Embryo Molecular Biology, National Health Commission and Shanghai Key Laboratory of Embryo and Reproduction Engineering, Shanghai, P.R. China; 9 Department of Neurology, Jiangbin hospital, Guangxi Zhuang Autonomous Region, Nanning, Guangxi, P.R. China

**Keywords:** factor related to cardiovascular health (FCH), longevity, genetic variation, lipid metabolism, Chinese

## Abstract

Cardiovascular disease (CVD) is one of the most important causes of human death, but no attention has been paid to cardiovascular health genes related to healthy longevity. Therefore, we developed a cohort study to explore such genes in healthy, long-lived Chinese subjects. A total of 13275 healthy elderly people were enrolled, including 5107 healthy long-lived individuals and 8168 age-matched control individuals with low CVD risk. Using a combination of whole-exome sequencing (WES) and genome-wide association studies (GWAS), we identified 2 genetic variants (TFPI rs7586970 T, p=0.013, OR=1.100. ADAMTS7 rs3825807 A, p=0.017, OR=1.198) associated with healthy lipid metabolism and longevity. Furthermore, we showed that an interaction among TFPI rs7586970, ADAMTS7 rs3825807 and APOE ɛ3 maintained normal blood lipid levels in centenarians by stratified analysis of CVD risk factors. Finally, through biological function analysis, we revealed clues regarding the mechanism of factor related to cardiovascular health (FCH) such as lipids and longevity. Kyoto Encyclopedia of Genes and Genomes (KEGG) pathway analysis indicated that the two variants above may be associated with longevity via FCH lipid metabolism pathways. From a meta-analysis of venous thrombosis patients, we unexpectedly found that rs7586970 T is associated with both longevity and protection against vascular disease.

## INTRODUCTION

Longevity is a complex biological phenomenon that is closely linked to a combination of multiple genes and various environmental factors. There are many genes and interrelated signaling pathways associated with longevity, but the molecular determinants of longevity are still unknown. Thus, the mechanisms of longevity are still an unsolved mystery.

Previous studies have demonstrated that the human life span may be regulated by genetic variation [1]. The genetic heritability of life span is approximately 25% in the general population but increases to 40% past 85 years of age and may be especially high in the long-lived population [2]. Longevity usually shows familial aggregation, with long-lived parents tending to have long-lived children. Therefore, genetic variation may be the most crucial factor leading to longevity. Several longevity-related variants have been identified by a traditional candidate gene approach and genome-wide association studies (GWAS) [3, 4]. Some longevity-related variants, like APOE [5], FOXO3 [6], CETP [6], IGFBP-3 [7] and SIRT1 [8], are concentrated in genes related to cardiovascular health metabolic pathways. Among them, the ones most closely correlated with longevity are regulatory genes involved in balancing lipid metabolism (such as APOE and CETP) [9]. Long-lived people tend to be able to maintain a good lipid metabolic balance, which may be an important reason for their long life.

Our previous studies found that certain variants of regulatory genes responsible for lipid metabolism balance (FOXO3 [4], APOE [10], CETP [4, 10], ND5 [11], HLA-DQB1 [12], etc.) could improve factor related to cardiovascular health(FCH) such as lipid metabolism balance and were associated with longevity in the population of Guangxi. APOE, as a recognized longevity-associated gene, reduces blood cholesterol levels by binding to specific lipoprotein receptors such as the low-density lipoprotein receptor (LDLR) [13]. The APOE genotype (Ɛ2, Ɛ3 and Ɛ4) is associated with healthy aging and longevity in Caucasian and Spanish populations [14]. Among long-lived people in Guangxi, China, APOE Ɛ3/ Ɛ3 is the protective genotype. In the elderly population, the total cholesterol and low-density lipoprotein levels of people with the APOE Ɛ3/ Ɛ3 genotype were lower than those of APOE Ɛ4 carriers [15]

Meanwhile, some variants of genes regulating lipid metabolism balance are also related to the risk factors of cardiovascular disease (CVD), diabetes, and dyslipidemia [16, 17]. Notably, TFPI rs7586970 T/C and ADAMTS7 rs3825807 A/G are both coronary artery disease (CAD) risk SNPs (p=9.00E^-6^, p=1.00E^-12^) [18].

TFPI is a circulating Kunitz-type protease inhibitor that acts as a natural anticoagulant and reduce risk occurrence probability of atherosclerotic plaques [19]. TFPI deficiency shows a greater burden of atherosclerosis in atherosclerotic senile (ApoE −/−) mice [20, 21]. In healthy middle-aged men, plasma free TFPI concentrations were significantly correlated with total cholesterol, LDL, triglyceride and apolipoprotein B levels [21]. TFPI can bind to some cell surface receptors, such as LDL receptor (LDLR)-associated proteins (known as Low Density Lipoprotein Receptor Associated Protein, LRP), to form lipoprotein-associated coagulation inhibitor (LACI) through their own Kunitz-type structure domain and GPI at C terminus region. Then, TC and LDL levels can be reduced [22–24], thereby inhibiting the thrombosis of atherosclerotic plaques and reducing the load of atherosclerotic plaques. TFPI rs7586970 T/C occurs at the glycosylphosphatidylinositol (GPI) anchor of tfpi-β protein, which can lead to the failure of tfpi-β and vascular endothelial cell anchoring [22].

ADAMTS7 belongs to the metalloproteinase family. Activated ADAMTS7 through its pro-domain cleavage hydrolyzes thrombospondin-5 (TSP5), an extracellular protein presenting such tissues as vascular walls and cartilages [25, 26]. ADAMTS7 promotes VSMC migration by degrading TS-5 [26]. Because VSMC migration is an important process in atherogenesis, it is likely that ADAMTS7 can also a contrary role in the development of atherosclerosis, the pathology underlying the vast majority of CAD. ADAMTS7 rs3825807 A/G, that the serine (Ser)-to-proline (Pro) substitution, hindered ADAMTS7 pro-domain cleavage, which reduces TSP5 hydrolysis. So, rs3825807 G/G genotype reduced migratory ability of vascular smooth muscle cells (VSMCs), intervening their ability to recognize and phagocytize oxidized LDL (Ox-LDL) to form fatty streaks [27]. We postulated that VSMCs with the ADAMTS7 rs3825807 A variant may migrate into the endothelium of subcutaneous vessels, phagocytize oxidized LDL, and prevent the occurrence of atherosclerosis. Previous studies have shown that ADAMTS7 overexpression in chondrocytes upregulates TNF-α [28, 29] and activates PDGFR-β enzyme activity. The combination of PDGF and PDGFR-β can result in VSMC migration in the MAPK pathway [30].

However, our study aimed to discover whether these risk or protective variants related to CVD in the population also have positive or negative effects associated with longevity. We assumed that these protective variants related to CVD may be the main causal determinants of a long and healthy life. Therefore, we developed a population study in healthy, long-lived Chinese subjects to explain the genetic mechanism of human longevity.

Previous studies in Bama have shown that the crude rate of cardiovascular and cerebrovascular events in elderly people over 90 years old is only 5.6% [31], while the incidence of cardiovascular disease in general elderly people over 65 years old has exceeded 20% [32]. What cause it big differences among them? One important reason for the longevity and low CVD prevalence of long-lived elderly people is that they have obvious advantages for keeping cardiovascular health such as, regulating blood lipids and blood glucose. This may be related to the presence of specific genetic characteristics, such as longevity gene-related variations or mutations in disease susceptibility sites, in healthy long-lived people. Specific variants may reduce the risk of cardiovascular disease by reducing risk genotypes and risk phenotypes.

Longevity and health are the best outcome variables for studying the causal relationship between longevity and cardiovascular disease risk. Therefore, multicohort and multi-omics studies were conducted to investigate the association between longevity-related genotypes and cardiovascular health phenotypes. The physiological and biochemical evidence of correlation between longevity and genetic variation was obtained by detecting and comparing the differences between long-lived individuals and non-long-lived, naturally aging elderly people in terms of gene variation, serum metabolic index level and phenotypic characteristics of longevity.

Meanwhile, combined with the phenotype analysis of multiple groups of cardiovascular health and long-lived populations, the interaction between longevity-related gene variants and health and longevity risk factors (obesity, hyperlipidemia, hyperglycemia) in healthy, long-lived populations can be used to preliminarily explore the mechanism of genetic longevity and confirm the special modes of interaction among multiple variants. This inquiry is of great significance in guiding the general population on how to effectively reduce the risk of disease, prolong life, improve quality of life, and achieve healthy aging.

## RESULTS

### Identifying longevity-related genetic variants

### Discovery strategy

**Figure 1:**
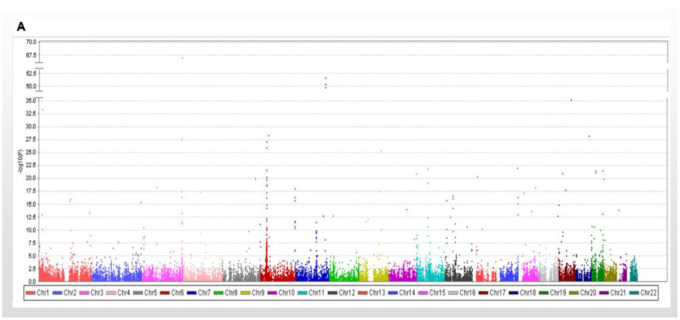
**Manhattan plot showing the results of the association with longevity.** Manhattan plot showing the results of the association with longevity in WES database.

The discovery strategy included 495 people with high longevity (mean age 101.2 ± 4.04 years). Among them, a total of 100 people (longevity group 1) were subjected to WES, including 17 males and 83 females. This group comprised 28 centenarians and 72 nonagenarians, with an average age of 96.9±4.17 years. They were from the Longevity and Health of Aging Population (LHAP) study conducted in Bama County, Guangxi, China, in 2008. For analysis of WES data and detection of sequencing quality, UCSC hg19 was used as the reference genome for sequence splicing, and meaningful mutation sites were identified. A total of 115,327 SNVs were found in the WES data, of which 84,914 were included in the database and 30,413 were not. There were 103,840 mutations in exons, 9618 in introns, and 2088 in intergenic regions. There were 50,289 missense mutations, 688 nonsense mutations, 41662 synonymous mutations, and 49 termination codon deletion mutations (Figure 1).

Another 395 longevity GWAS data analyzed in the present study are from the CLHLS on longevity, aged (102.3±3.18) years (longevity group 2). All of the subjects were Han Chinese. The CLHLS GWAS has the largest worldwide sample size of centenarians, 2.7 times as large as the next largest sample of GWAS on longevity in centenarians. The CLHLS GWAS included 5.6 million SNPs for each of the centenarians and controls and followed the Strengthening the Reporting of Genetic Association Studies (STREGA) reporting guidelines for GWAS quality control, including genotyping errors, population stratification and HWE, with a full quality item score of 12, indicating good quality and completeness. The 94% typing rate in the population was more than 90% for a total of 818048 SNPs [1]. p<10^-4^ was used as a significant criterion [33–35] to compare the 1000 Genomes Project Chinese population (control group 1; including CHS, CHB, and CDX populations; 301 people; age <60 years old), and 56 mutations were selected as candidate mutations (Supplementary Table 1).

### Replication strategy

Mutations shared between WES and GWAS datas were considered the final candidate variants. Two SNPs (rs7586970, p_genotype-TT_=4.09E-04; rs3825807, p_genotype-AA_=0.05) were replicated and validated in 5107 long-lived elderly individuals (longevity group, age ≥90 years) and 8469 control individuals (control group). There was a difference in distribution between the two groups, and the rs7586970 T allele and rs3825807 A allele frequency were significantly increased in the longevity group and positively correlated with longevity (p_allele-T_=0.013, OR=1.100. p_allele-A_=0.017, OR=1.198) (Table 1, Supplementary Table 2).

**Table 1:** Variants associated longevity.

		**Major allele Case/Control**	**Minor allele Case/Control**	**p**	**OR**	**95%CI**	**Major homo Case/Control**	**Hetro Case/Control**	**Minor home Case/Control**	**p**
Longevity vs. Controls	rs7586970	8695/14379	1181/2149	0.013	1.100	1.02-1.187	3801/6256	44/141	1093/1867	4.09E-04
	rs3825807	2100/4447	278/705	0.017	1.198	1.033-1.389	929/1918	18/47	242/611	0.050
Centenarians vs. Nonagenarians	rs7586970	3298/5397	386/795	4.69E-04	1.259	1.106-1.432	1466/2335	10/34	366/727	0.001
	rs3825807	1109/991	113/165	1.37E-04	1.634	1.267-2.107	503/426	5/13	103/139	7.51E-04
Centenarians vs. Controls	rs7586970	3298/14379	386/2149	2.9E-05	1.277	1.138-1.432	1466/6256	10/141	366/1867	2.0E-05
	rs3825807	1109/4447	113/705	3.1E-05	1.556	1.262-1.918	503/1918	5/47	103/611	1.61E-04
Nonagenarians vs. Controls	rs7586970	5397/14379	795/2149	0.745	1.015	0.930-1.107	2335/6256	34/141	727/1867	0.045
	rs3825807	991/4447	165/705	0.599	0.952	0.793-1.143	426/1918	13/47	139/611	0.777

The longevity group contained 1859 centenarians aged 102.8 ± 2.62 years and 3248 nonagenarians aged 93.9 ± 2.64 years. In a comparison with the nonagenarian group, two SNPs were recognized in the centenarian group (rs7586970, p_genotype_=0.001; rs3825807, p_genotype_=7.51E-04); the frequency of the rs7586970 T allele (p_allele_=4.69E-4, OR=1.259) and rs3825807 A allele (p_allele_=1.37E-04, OR=1.634) showed a significant increase in the centenarian group, and both were positively correlated with longevity. Compared with the control group, the rs7586970 and rs3825807 distributions had significant differences in the centenarian group. The frequency of rs7586970 T and rs3825807 A also increased significantly, and both were positively correlated with longevity (p_allele-T_=2.90E-5, OR=1.277. p_allele-A_=1.37E-04, OR=1.634). In the nonagenarian group compared with the control group, only rs7586970 showed a difference in distribution between the two groups (p_genotype-TT_=0.04) (Table 1, Supplementary Table 2).

### Mutation interaction analysis

### Analysis of the interaction between the mutations and the longevity-associated gene APOE

The APOE gene, a lipid metabolism balance gene, is currently the only recognized longevity-associated gene [36]. APOE has three common alleles: ε2, ε3, and ε4. Our study found that APOE ε3/ε3 is a protective genotype in the longevity population in Guangxi, which has a positive effect on longevity. Comparing the centenarian group and the nonagenarian group revealed significant differences in the frequency of rs7586970 and rs3825807 genotypes among APOE ε3 allele carriers (p=4.28E-04, p=0.009). The rs7586970 A allele and rs3825807 T allele were positively correlated with longevity (p_allele_=9.70E-05, OR=1.303. p_allele_=8.91E-04, OR=1.575). In the centenarian population, APOE ε3 cooperative interacts with TFPI rs7586970 and ADAMTS7 rs3825807 (Table 2).

**Table 2:** Interactive analysis of TFPI rs7586970, ADAMTS7 rs3825807 between APOE ε3.

	**APOE ε3**								
rs7586970	TT	CC	TC	P	T	C	P	OR	95%CI
Centenarians	1232	20	319		2783	359			
Nonagenarians	1999	67	647	4.28E-04	4645	781	9.7E-05	1.303	1.141-1.490
rs3825807	AA	GG	AG	P	A	G	P	OR	95%CI
Centenarians	406	5	90		902	100			
Nonagenarians	369	12	127	0.009	865	151	8.91E-04	1.575	1.203-2.061

### Analysis of interactions between mutations

MDR analysis showed that the cross-validation consistency of the rs7586970 and rs3825807 models was 10/10 (p = 3.00E-4), comparing the nonagenarian group with the centenarian group. There was an cooperative interaction between rs7586970 and rs3825807 (Table 3).

**Table 3:** Multifactor dimensionality reduction (MDR) interactive analysis of TFPI rs7586970 between ADAMTS7 rs3825807.

**Model**	**Training Balanced accuracy**	**Testing Balanced accuracy**	**p**	**Cross-validation Consistence**
Centenarians vs Nonagenarians				
rs3825807	0.5725	0.5720	3.00e-4	10/10
rs7586970 rs3825807	0.5732	0.5721	3.00e-4	10/10

In addition, our study found that, compared with that of the control, the TTAA frequency in centenarians was significantly higher than TTGG, showing a significant difference. Individuals with the TTAA haplotype were more likely to live longer (p=0.001, OR=1.57) (Table 4).

**Table 4:** Combined effects of TFPI rs7586970 *ADAMTS7 rs3825807 in Centenarians.

**Genotype**	rs7586970 TT * rs3825807 AA	rs7586970 TT * rs3825807 GG			
Group	Centenarians/Control	Centenarians/Control	P	OR	95%CI
Number	423/1484	73/402	0.001	1.57	1.196-2.060

### Phenotype information of subjects

### Centenarian group vs. nonagenarian group

There were significant differences in FBG. The levels of FBG in the centenarian group were significantly higher than those in the nonagenarian group (p<0.05). There were no differences in gender, BMI, TC, TG, or LDL-C between the centenarian group and the nonagenarian group.

### Nonagenarian group vs. control group

There were significant differences in BMI. The BMI value in the nonagenarian group was significantly higher than that in the control group (p<0.05). There were no differences in gender, FBG, TC, TG, or LDL-c between the nonagenarian group and the control group.

### Centenarian group vs. control group

There were significant differences in age, FBG and TG. The levels of FBG and TG in the nonagenarian group were significantly higher than those in the control group (p<0.05). There were no differences in gender, BMI, TC or LDL-c between the centenarian group and the control group.

### Longevity group vs. control group

There were significant differences in age, BMI, FBG and TG (p<0.05). The levels of BMI, FBG and TG in the longevity group were significantly higher than those in the control group. There were no differences in gender, TC, or LDL-c (Supplementary Table 3).

### Identification of associations between variant polymorphisms and metabolic phenotypes

A comparison between centenarians and nonagenarians found that the polymorphism distribution of rs7586970 in the group with normal BMI, FBG, TC, TG and LDL levels was statistically significant between the different age groups (p<0.05). Comparing the centenarians with the control group, the polymorphism distribution of rs7586970 in the group with normal BMI, FBG, TC, TG and LDL levels was also different between the two age groups. Then, the nonagenarian, longevity and control groups were compared, and the results showed no difference in the distribution of the rs7586970 polymorphism among the age groups with normal BMI, FBG, TC and LDL levels. Therefore, the rs7586970 polymorphism was correlated with normal BMI, FBG, TC and LDL in different age groups (Supplementary Table 4).

Similarly, a comparison between centenarians and nonagenarians found that the difference in the polymorphism distribution of rs3825807 was statistically significant between the different age groups in the group with normal BMI, FBG, TC, TG and LDL levels and the group with BMI≥23 kg/m^2^ (p<0.05). Compared with the control group, the polymorphism distribution of rs3825807 was also different in the group with normal BMI, FBG, TC, TG and LDL levels and the group with BMI≥23 kg/m^2^. Comparing the nonagenarians with the control group, there was no difference in the rs3825807 polymorphism distribution between the age groups in the normal BMI, FBG, and TC level group and the BMI≥23 kg/m^2^ group. Therefore, the polymorphism distribution of rs3825807 was correlated with normal levels of FBG and TC and different BMI values in different age groups (Supplementary Table 4).

### Stratification analysis of variant polymorphisms and metabolic phenotypes

To eliminate the influence of confounding phenotype information on the association between each variation and phenotype, our study analyzed the relationships between frequency of allele variation, genotype, haplotype and lipids (-/+) +FBG (-/+) +BMI (-/+) according to the above grouping criteria in Materials and Methods.

To eliminate the influence of two alleles in one variant, we performed a stratified analysis of the relevance between alleles and metabolic phenotypes. The results showed that rs7586970 T is specifically correlated with the metabolic level of lipids (-) +FBG (-) +BMI (+) in centenarians. The rs7586970 T allele was specifically correlated with normal blood lipid and glucose metabolism levels in centenarians (Figure 2A, Supplementary Table 5).rs3825807 A showed a specific correlation with normal levels of lipids, FBG and BMI in centenarians (Figure 2B, Supplementary Table 5), and thus, rs7586970 T and rs3825807 A may represent a protective genetic marker of normal blood lipid and glucose levels in centenarians.

**Figure 2:**
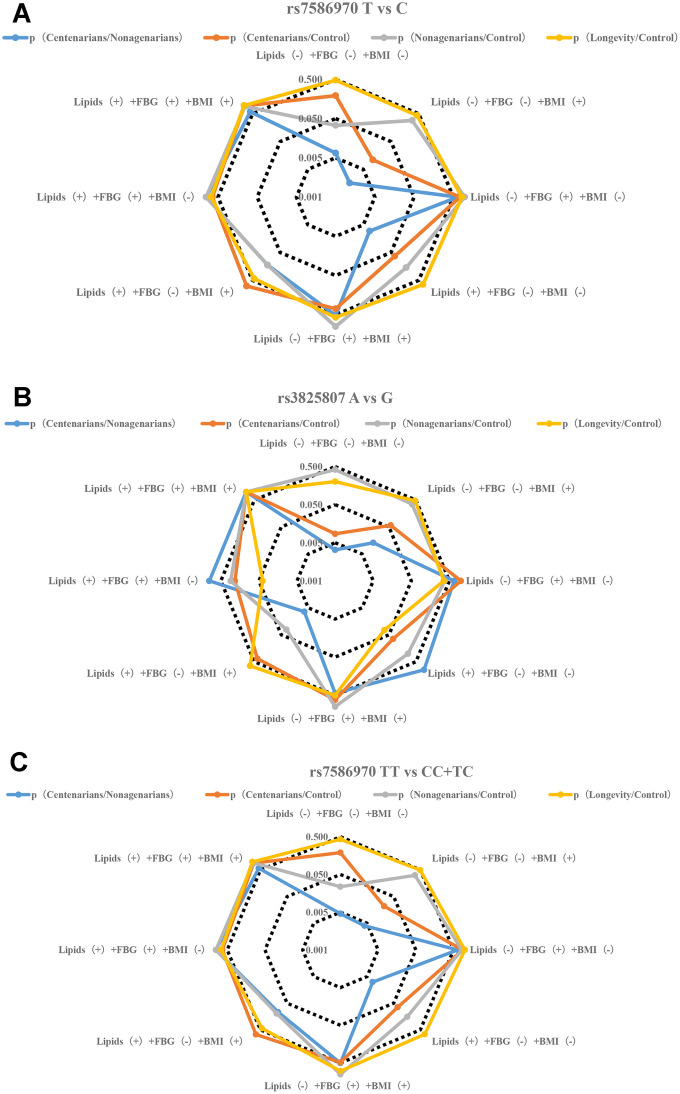
**Stratification analysis of metabolic phenotype of TFPI rs7586970 and ADAMTS7 rs3825807.** (**A**) Correlation analysis of allele frequency of TFPI rs7586970 with metabolic phenotype. The frequency of the rs7586970 T is specifically correlated with the metabolic level of lipids (-) +FBG (-) +BMI (+) in centenarians. The allele frequency of rs7586970 T was significantly increased in the centenarians group. (**B**) Correlation analysis of allele frequency of ADAMTS7 rs3825807 with metabolic phenotype. The frequency of the rs3825807 A is specifically correlated with the metabolic level of lipids (-) +FBG (-) +BMI (-) in centenarians. (**C**) Correlation analysis of genotype frequency of TFPI rs7586970 with metabolic phenotype. The rs7586970 recessive model (TT/TC+CC) showed a specific correlation with lipids (-) +FBG (-) +BMI (+) in centenarians.

**Figure 2:**
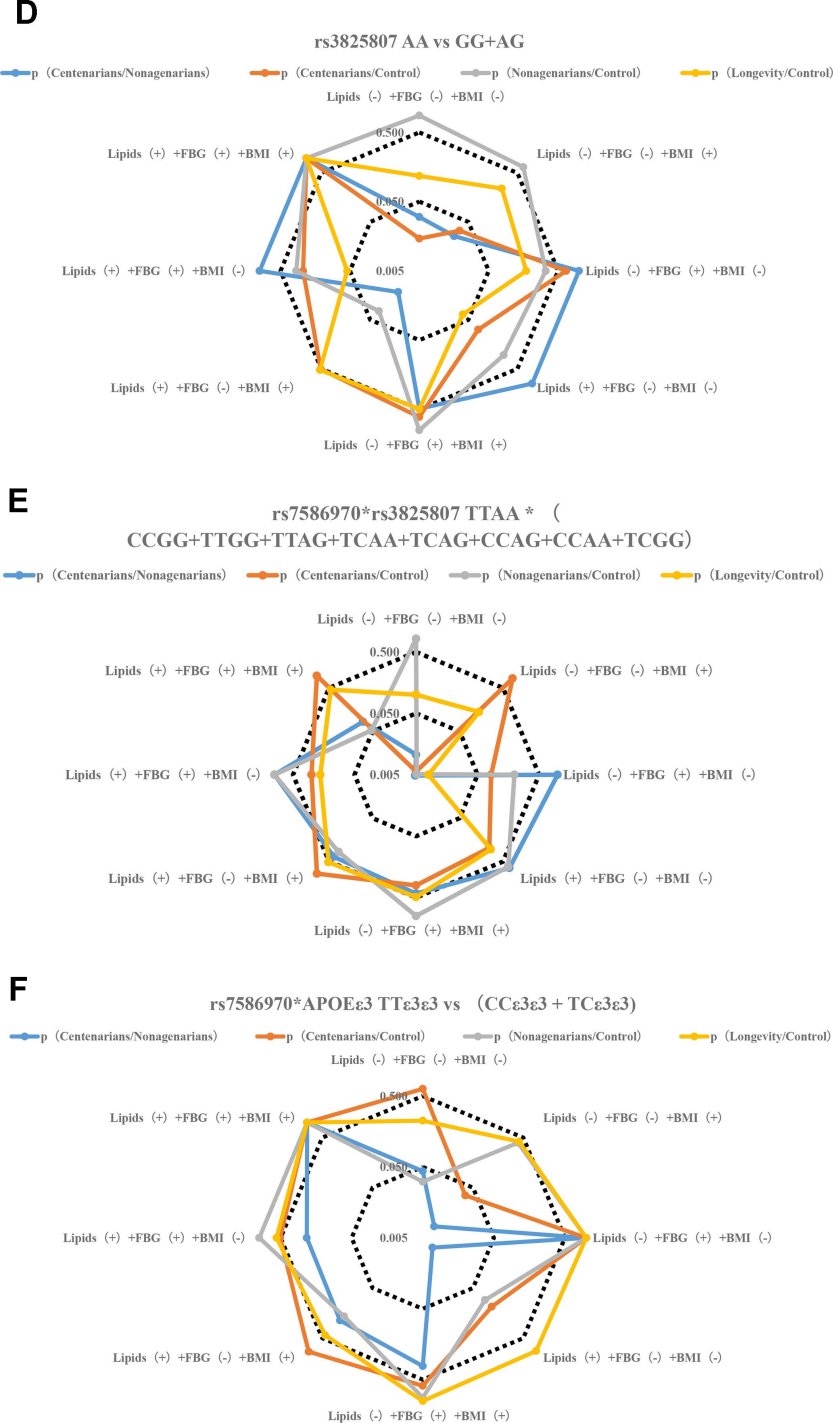
**Stratification analysis of metabolic phenotype of TFPI rs7586970 and ADAMTS7 rs3825807.** (**D**) Correlation analysis of genotype frequency of ADAMTS7 rs3825807 with metabolic phenotype. The rs3825807 recessive model (AA/AG+GG) showed a specific correlation with lipids (-) +FBG (-) + BMI (-/+) in centenarians. (**E**) Correlation analysis between the interaction of TFPI rs7586970 and ADAMTS7 rs3825807 with metabolic phenotype. TTAA has a specific and significant correlation with lipids (-) +FBG (-) +BMI (-) in centenarians. (**F**) Correlation analysis between the interaction of TFPI rs7586970 and APOE ε3 with metabolic phenotype. TTε3ε3 was specifically correlated with lipids (-) +FBG (-) +BMI (+) in centenarians.

**Figure 2:**
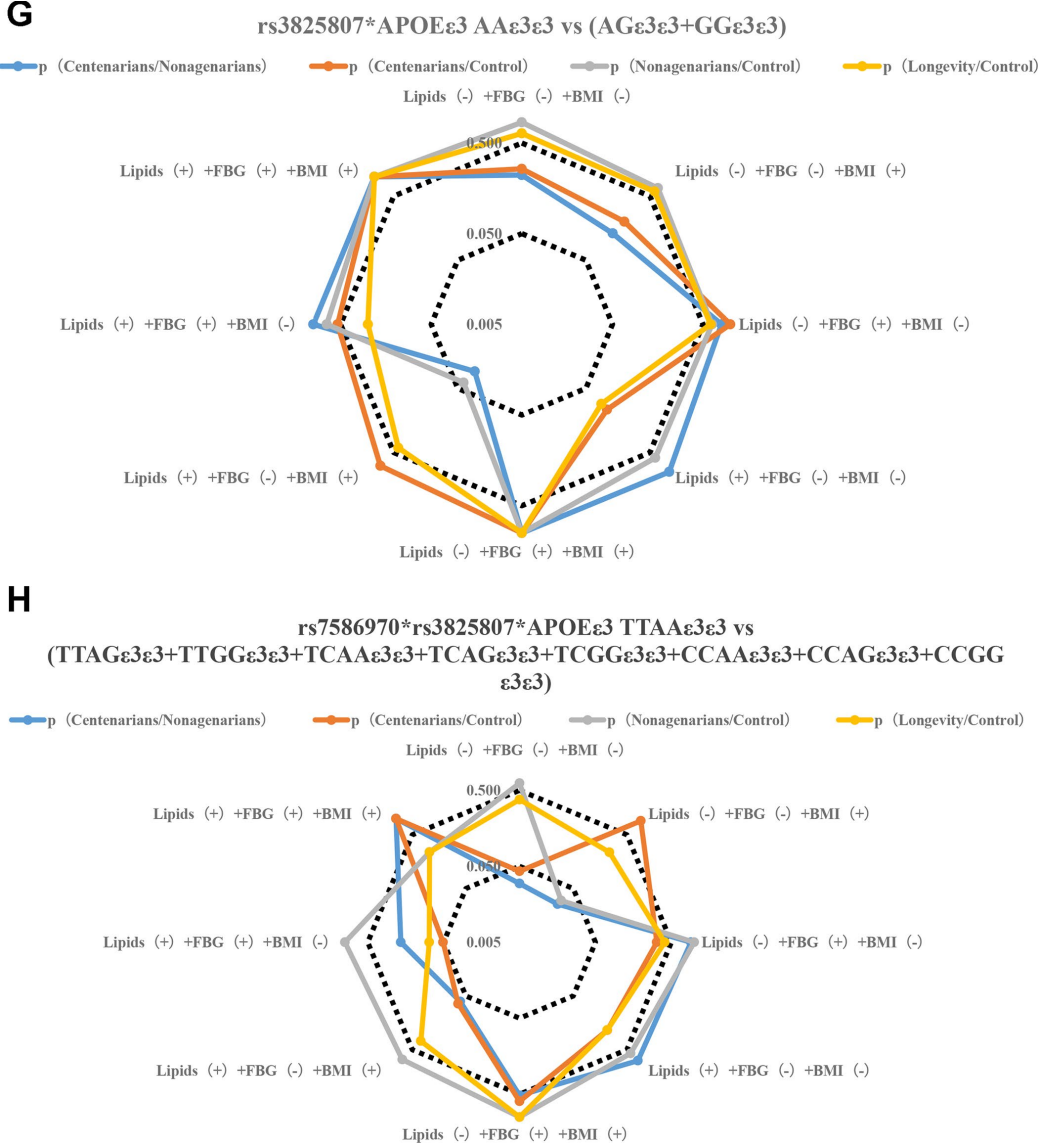
**Stratification analysis of metabolic phenotype of TFPI rs7586970 and ADAMTS7 rs3825807.** (**G**) Correlation analysis between the interaction of ADAMTS7 rs3825807 and APOE ε3 with metabolic phenotype. AAε3ε3 was not specifically correlated with BMI, blood glucose and lipid in centenarians. (**H**) Correlation analysis between interaction of TFPI rs7586970, ADAMTS7 rs3825807 and APOE ε3 with metabolic phenotype. AATTε3ε3 in the centenarians group showed a specific correlation with lipids (-) +FBG (-) +BMI (-).

To clarify the relationship between the variation in the population and different levels of metabolism, a gene model was used to analyze the correlations between variant genotypes and metabolic phenotypes. A recessive model (TT/TC+CC) was used to analyze the relationship between the genotype of TFPI rs7586970 and metabolic phenotype. The rs7586970 recessive model (TT/TC+CC) showed a specific correlation with lipids (-) +FBG (-) +BMI (+) in centenarians (Figure 2C, Supplementary Table 5). The rs3825807 recessive model (AA/AG+GG) showed a specific correlation with lipids (-) +FBG (-) + BMI (-/+) in centenarians (p<0.05, OR>1) (Figure 2D, Supplementary Table 5).

This study analyzed the correlation between the haplotypes of two variants and a metabolic phenotype and then analyzed whether there is cooperative interaction between the two variants in the process of affecting blood glucose and blood lipids. Based on the above study, the recessive models of two variants are correlated with blood glucose, blood lipids and BMI. Due to the influence of sample size and to reduce the impact of heterozygous haplotypes with TT and AA on metabolic phenotype, our study combined the heterozygous haplotypes and focused on comparing the overall relationship of TTAA and other heterozygous haplotypes(CCGG+TTGG+TTAG+TCAA+TCAG+CCAG+CCAA+TCGG) with the level of blood glucose, blood lipids and BMI of each group. The result shows that TTAA had a specific and significant correlation with lipids (-) +FBG (-) +BMI (-) in centenarians (p<0.05. OR>1) (Figure 2E, Supplementary Table 6).

The results of a previous analysis indicate that APOE ε3, TFPI rs7586970 T and ADAMTS7 rs3825807 A have synergistic interactions among centenarians. We compared whether differences in the frequency of TTε3ε3 and (CCε3ε3+TCε3ε3), AAε3ε3 and (GGε3ε3+AGε3ε3) were correlated with the metabolic phenotype in different populations. The result shows that TTε3ε3 was specifically correlated with lipids (-) +FBG (-) +BMI (+) in centenarians. TTε3ε3 was correlated with high BMI and maintained normal levels of blood glucose and lipids in centenarians (Figure 2F, Supplementary Table 7). ADAMTS7 rs3825807 AA had no interaction with APOE on the regulation of BMI, blood glucose and lipids in centenarians (Figure 2G, Supplementary Table 7).

We also compared whether differences in the frequency of AATTε3ε3 and (AGTTε3ε3+GGTTε3ε3+AATCε3ε3+AGTCε3ε3+GGTCε3ε3+AACCε3ε3+AGCCε3ε3+GGCCε3ε3) were correlated with the metabolic phenotype in different populations. The result of research shows that AATTε3ε3 in the centenarian group showed a specific correlation with lipids (-) +FBG (-) +BMI (-) (Figure 2H, Supplementary Table 8).

### Bio-functional analysis

### Joint prediction of variant effects on gene functions by SIFT and PolyPhen 2 software

SIFT and PolyPhen 2 were used to predict the potential functional effects of the rs7586970 and rs3825807 variants on TFPI and ADAMTS7, respectively. PolyPhen 2 analysis results show that the effects of rs7586970 and rs3825807 variations on TFPI and ADAMTS7 are benign and do not affect protein function (Supplementary Figure 1A, 1B). However, SIFT software analysis results show that the intersection number of N and S at position 221 of the rs7586970 variation is 0.00, so the rs7586970 variation injures the function of the TFPI protein. The intersection number of S and P at the 214 position of the ADAMTS7 variation was 0.27, so the rs3825807 variation did not influence the function of the ADAMTS7 protein (Supplementary Figure 1C, 1D).

### Motifs analysis and prediction of splicing factors (SFmap) software predicts transcriptional splicing results of TFPI rs7586970 and ADAMTS7 rs3825807

SFmap was used to analyze the potential role of variants in the process of transcriptional splicing in the genes. The results showed that both the TFPI rs7586970 C and ADAMTS7 rs3825807 G variants affected the intrinsic splicing of the pre-mRNAs of the genes in which the variants were located. The TFPI rs7586970 C variant disrupts the binding site of Tra2β (Transformer-2 protein homolog beta) and PTB (polypyrimidine tract binding protein polypyrimidine tract binding protein) and changes the splice position of the splicing factor in SRp20. The ADAMTS7 rs3825807 G variant changes the splicing position of hnRNPF (Heterogeneous nuclear ribonucleoproteins F) and hnRNPA2B1 (Heterogeneous nuclear ribonucleoproteins A2B1) (Supplementary Figure 2).

### Results of spatial structural prediction of mutant proteins

The SWISS-MODEL prediction results showed that the TFPI rs7586970 T/C variation replaced Asn with Ser at position 221. The T terminal structure of the amino acid in TFPI rs7586970 C is different from that of TFPI rs7586970 T. Comparing the structure of the two variants with PyMOL showed a slight difference in the structure of the variants (RMS=0.004 (306 to 306 atoms)). Thus, TFPI rs7586970 T/C alters the spatial structure of the protein.

The ADAMTS7 rs3825807 A/G variation replaced Ser with Pro at position 214. The A terminal structure of the amino acids in ADAMTS7 rs3825807 G is different from that of ADAMTS7 rs3825807 A. Comparing the structure of two variants by PyMOL, the results showed a signifi-cant difference in the structure of variants (RMS=0.004 (306 to 306 atoms)). ADAMTS7 rs3825807 A/G also changes its spatial structure (Supplementary Figure 3).

### Results of IMP analysis

The results showed that the TFPI gene interacted indirectly with the ADAMTS7 gene through the SPARC gene and PDGFRB gene. TFPI gene interacts indirectly with the APOE gene via DAB2 and LRP1. ADAMTS7 interacts indirectly with the APOE gene via PDGFRB (Supplementary Figure 4, Supplementary Results 6.4).

### KEGG signal pathway analysis

Combined with the analysis results of IMP and MDR software, a total of 21 genes interacted with TFPI and ADAMTS7. Annotating the signaling pathways involved in these genes in the KEGG database showed that most of the genes were involved in cholesterol metabolism, the MAPK signaling pathway, the PI3K-Akt signaling pathway, and the AGE-RAGE signaling pathway in diabetic complications or other metabolic-related pathways (Supplementary Figure 5). Based on a comprehensive analysis of the above phenotypic stratification results, we believe that TFPI rs7586970 and ADAMTS7 rs3825807 may be associated with healthy longevity by affecting lipid metabolism balance (Supplementary Figure 6).

### Meta-analysis

Previous studies have shown that the TFPI rs7586970 T/C mutation affects plasma total TFPI concentration. Decreased TFPI concentration will increase the risk of venous thromboembolism (VTE) and myocardial infarction (MI) [37, 38]. In this study, a total of 6 groups were included to analyze the influence of TFPI rs7586970 T/C variation on the risk of venous thrombosis caused by increased plasma TFPI concentrations [6, 39–43]. Meta-analysis results showed that among 1829 venous thrombosis patients and normal controls, plasma TFPI concentration was significantly reduced in carriers of the rs7586970 T allele (p=0.03, OR=0.87). Carriers of the rs7586970 T allele have a significantly reduced risk of venous thrombosis, which is a protective gene for genetic variation in the disease (Supplementary Figure 7, Supplementary Table 9).

## DISCUSSION

It is well known that imbalanced lipid metabolism is associated with age-related diseases such as metabolic syndrome, CVD and cerebrovascular disease, but there is still little published research on the relationship between cardiovascular health aging or longevity and the balance of lipid metabolism. Therefore, we developed a population-based study to identify cardiovascular health and longevity-associated genetic variants in long-lived subjects. We identified both TFPI rs7586970 T and ADAMTS7 rs3825807 A as longevity-related gene variants in Chinese for the first time (TFPI rs7586970, p_allele_=0.013, OR=1.100; ADAMTS7 rs3825807, p_allele_=0.017, OR=1.198). Furthermore, by comparing centenarians with controls, we showed that APOE ɛ3 and the two novel variants in our study population jointly increased the probability of healthy longevity (OR=1.570, p=0.001). Then, for lipid balance phenotypes, we observed that these variants (TFPI rs7586970 T and ADAMTS7 rs3825807 A allele) with or without APOE ɛ3/3 were significantly correlated with normal TC, TG, and LDL levels in centenarians (p=0.03, OR=2.25). We speculate that TFPI rs7586970 TT, ADAMTS7 rs3825807 AA and APOE ɛ3 are important independently or jointly for maintaining cardiovascular health lipid metabolism balance in the bodies of long-lived individuals. Along these lines, we showed that carrying both lipid metabolism balance variants has a greater positive effect on longevity than carrying either variant alone.

**Figure 3:**
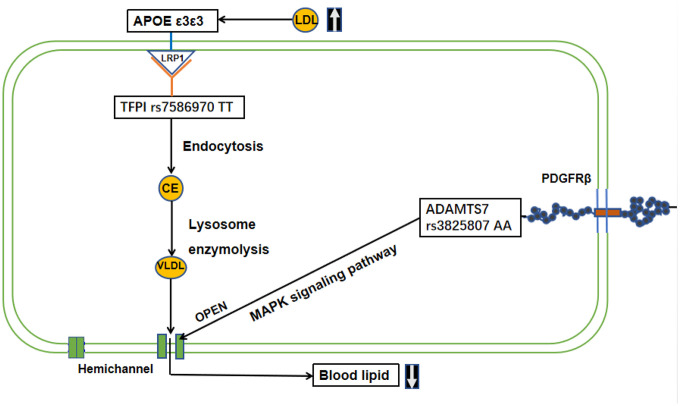
**Mechanism or interactive pathway of TFPI, ADAMTS7 and APOE in lipid metabolism homeostasis in longevity.** TFPI rs7586970 TT, with normal function, can be formed lipoprotein-associated coagulation inhibitor (LACI) through C-terminal region glycophosphatidylinositol (GPI) anchoring point by binding to LDL receptor (LDLR)-related proteins, known as Low-Density Lipoprotein Receptor Associated Protein 1(LRP1). APOE also regulates blood cholesterol levels by binding to LDLR. When the blood lipid concentration increases, APOE transport of lipids by binding to LRP1. Then, TFPI rs7586970 TT interacts with APOE via the LRP1to carry out endocytosis. After that convert lipid to cholesterol ester (CE). The CE is broken down into VLDL by enzymolysis in lysosomes. In the meantime, ADAMTS7 rs3825807 AA activates PDGFR-β enzyme activity to bond PDGF. The combination of PDGF and PDGFR-β can result in VSMC migration in the MAPK pathway. PDGFR-β is a typical tyrosine receptor kinase that activates downstream growth factors, such as receptor-binding protein 2 (Grb2), son of sevenless (Sos), Ras and other factors, activating the MAPK pathway and stimulating hemichannel opening to release VLDL into the extracellular environment or adjacent cells.

Hemichannel can be opened by PDGFR-β stimulus through MAPK signaling pathway (map04010, map04540). Previous studies have shown that ADAMTS7 overexpression in chondrocytes upregulates TNF-α [28, 29] and activates PDGFR-β enzyme activity. However, rs3825807 G/G genotype in vascular smooth muscle cells reduced their migratory ability, reducing their ability to recognize and phagocytize oxidized LDL (Ox-LDL) to form fatty streaks [39]. There, we postulated that vascular smooth muscle cells (VSMCs) of centenarians with the ADAMTS7 A variant may migrate into the endothelium of subcutaneous vessels, phagocytize oxidized LDL, stimulate hemichannel opening to release VLDL into the extracellular environment or adjacent cells, contributing to preventing the occurrence of atherosclerosis through the MAPK pathway.(Figure 3, Supplementary Figure 6).

Clearly, it is necessary to further verify the mechanism of spatial structural changes caused by these mutations through cell and animal experiments. However, it is more important to uncover why these mutations are present in the population and to explore how they are preserved. Our preliminary study confirmed that these variations were closely related to the living environment of the population. In our study, the centenarians who underwent WES were all from Bama, Guangxi, China. The people of Bama are noted for their exceptional longevity; the county ranks fifth in the world in that respect. Bama County has a centenarian population (rate of longevity) of 35 per 100,000, compared with 2.19 per 100,000 in the nation overall [44]. Notably, the longevity in the Bama area shows strong familial aggregation. Thus, the Bama area, with its relatively closed living environment, homogeneous genetic background and low proportion of immigrants, is an ideal place for longevity research, and the specific environment of the long-lived Bama population may be an important reason to preserve this protective mutation. In the process of continuous evolution, one of the two alleles of an SNP was a healthy variant and the other was a CAD risk variant; the healthy variant was significantly more common than the risk variant. Over the evolution of Bama's long-lived population, this variation, which promotes longevity and health, has been strongly selected, as it is conducive to human survival and reproduction. Therefore, the environment in which this protective variation has been preserved will be further explored in future work.

In general, we found that at the same locus, the mutant allele has a completely different impact from the wild-type allele in disease susceptibility and explains the significance of the protective allele in a healthy, long-lived population. Coexisting longevity promotion variants (TFPI rs7586970 T and ADAMTS7 rs3825807 A) and disease-onset risk variants (TFPI rs7586970 C and ADAMTS7 rs3825807 G) in healthy longevity may maintain the health (homeostasis) of the body through complex multigene networks.

Longevity is a polygenic effect of biological phenomena. Long-lived, healthy individuals who carry disease-risk genes but do not develop disease may be experiencing an ectopic effect of other genes associated with health and longevity, a possibility that needs to be explored in the future.

Our preliminary study suggests that the regulatory interactions between the targeting enhancers and their target genes are particularly important in linking non-coding risk variants from genome-wide association studies to candidate causal genes. Our studies found that variants in FCH lipid metabolism regulation genes in long-lived elderly people in Guangxi may be involved in maintaining the balance of lipid metabolism, thereby delaying the occurrence of diseases and promoting longevity [4, 10–12]. The study further confirmed that long-lived individuals in Guangxi, especially centenarians, have a lower incidence of aging-related diseases than the local general population [45]. Recent studies also have shown that there is a high density of genetic variation in the enhancer region [46–48]. Disease-related SNPs occur in non-coding parts of the genome more than 90% of the time [49].

These phenomena suggest that the pathway of SNPs involvement may disrupt the binding sites of transcription factors at the enhancer, alter the sequence of enhancer RNAs, and thus may disturb the important functions of cells. The prospect has led us to a new understanding of the relationship between disease, health and longevity. There may be a specific mode of interaction between genetic variation in human health as shown by longevity and genetic variation in disease risk. Interactions among multiple genes jointly maintain the health status of the body and can even be beneficial for longevity. This paradigm provides a new idea of how to evade the internal environmental factors (genes) of disease risk and how to reasonably prevent complex multigene diseases.

## MATERIALS AND METHODS

### Subjects

**Figure 4:**
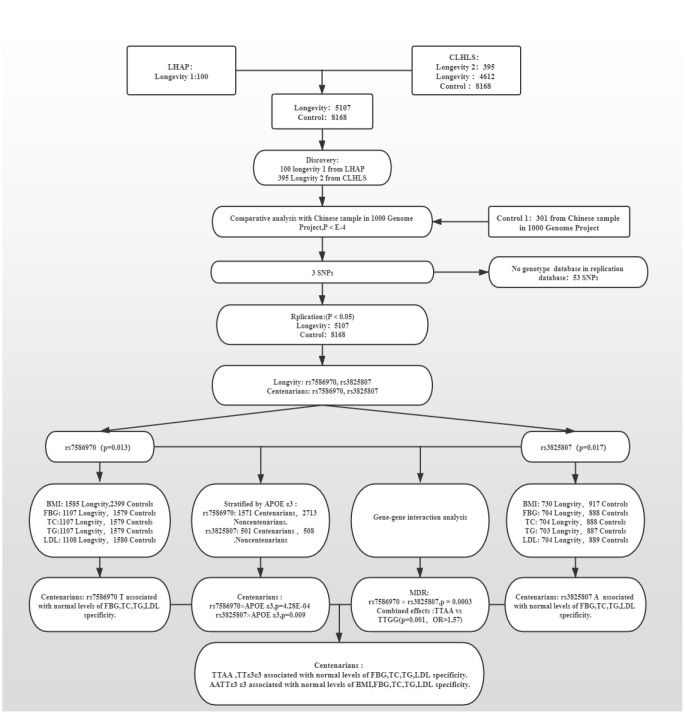
**A flow chart of the consecutive analysis steps.**

We conducted a case-control study. A flow chart of the consecutive analysis steps is depicted in Figure 4 (Supplementary Materials and Methods 1. Subjects).

### WES and quality controls

Genomic DNA was isolated from peripheral blood leukocytes by standard methods [50] (Supplementary Materials and Methods 2. WES and quality controls).

### GWAS genotype and quality controls

The population for the GWAS in this study is from CLHLS. All of the subjects were Han Chinese (Supplementary Materials and Methods 3. GWAS genotype and quality controls).

### Discovery-evaluation strategy

We combined the variants data from exon sequencing and GWAS and compared them with the 1KGP III Chinese population (control group 1, including the CHS, CHB, and CDX populations; 301 people in all, age <60 years old), selecting the modest a priori discovery threshold of P<10^-4^ [33–35]. The common variations were replicated and verified in 5107 long-lived individuals (longevity group) and 8469 non-long-lived elderly people (control group). Following the usual practice, we applied a nominal significance level of p<0.05 here as well as in phenotypic stratification analysis (Supplementary Materials and Methods 4. Discovery-evaluation strategy).

### Stratified analysis interaction between variants and APOE ɛ3

The APOE gene is currently recognized as a longevity-associated gene [51, 52] and its alleles include ɛ2, ɛ3 and ɛ4. APOE ɛ3 is positively correlated with healthy aging and longevity in Bama, Guangxi [36]. In this study, the Pearson chi-squared test or Fisher exact test was applied for stratified analysis of the interaction between APOE ɛ3 and other genotypes.

### Gene-gene interaction network analysis

Multifactor dimensionality reduction (MDR) software was used to identify the presence of interactions between genetic variants. We analyzed our experimental results in the functional context of gene-gene networks from multiple organisms in Integrative Multispecies Prediction (IMP). Functional gene networks were constructed in IMP, where genes connected by an edge in a functional network were predicted to participate in similar biological processes.

### Analysis of the interaction between variation and metabolic genotype

The enrolled samples were divided into four groups according to age: centenarians (age≥100 years), nonagenarians (90 years≤age<100 years), a combined long-lived group consisting of centenarians and nonagenarians (age≥90 years) and a control group (age<90 years). The phenotype information of this study is grouped as follows: (1) BMI (-): BMI<23 kg/m^2^. (2) BMI (+): BMI≥23 kg/m^2^ [53]. (3) Fasting blood glucose (FBG) (-): FBG<7.0 mmol/l. (4) FBG (+): FBG≥7.0 mmol/l [54]. (5) Lipids (-): TC≤5.72 mmol/L, TG≤1.7 mmol/L and LDL≤3.3 mmol/L. (6) Lipids (+): TC>5.72 mmol/L, TG>1.7 mmol/L and LDL>3.3 mmol/L [55, 56]. The associations of alleles, genotypes and haplotypes with phenotypes were analyzed separately by univariate analysis or multifactorial stratification analysis, as appropriate.

### Bioinformatics functional analysis

See Supplementary Materials and Methods 5. Bioinformatics functional analysis.

### Meta-analysis of the effect of TFPI rs7586970 T/C on plasma TFPI concentration

See Supplementary Materials and Methods 6. Meta-analysis of the effect of TFPI rs7586970 T/C on plasma TFPI concentration.

### Statistical analysis

Genotypes were evaluated for departure from Hardy–Weinberg equilibrium (HWE) in the controls using chi-squared tests. Variants with p<0.05 were considered to deviate from HWE. Minor allele frequency (MAF) of variants was used as the risk allele frequency. The genotype frequencies of the Chinese (CHS, CHB, CDX) population from the 1000 Genomes database were used as the references for selecting candidate SNVs. If the quantitative data were normally distributed, the t-test was used to compare mean groups. Nonnormally distributed data are tested using nonparametric test. Intergroup comparisons of qualitative data used the Kruskal-Wallis H. Comparisons of two groups were performed with the Pearson χ^2^ or Fisher exact test. The P value was corrected with the Bonferroni test. A two-sided p value<0.05 was considered statistically significant.

## Supplementary Material

Supplementary Materials and Results

Supplementary Figures

Supplementary Table 1

Supplementary Tables 2, 3 and 4

Supplementary Table 5

Supplementary Table 6

Supplementary Table 7

Supplementary Table 8

Supplementary Table 9
