# Supplementary material for: Identification of cardiovascular health gene variants related to longevity in a Chinese population
Source: Aging (Albany NY). 2020 Sep 7;12(17):16775–802. doi: 10.18632/aging.103396 (PMC7521493; doi:10.18632/aging.103396)
Supplement: Supplementary Materials and Results [file aging-12-103396-s006..pdf]

SUPPLEMENTARY MATERIALS

SUPPLEMENTARY MATERIALS AND METHODS

Subjects

A total of 13,275 Chinese people were included, comprising 5107 long-lived individuals (longevity group, age ≥90 years) and 8168 non-long-lived elderly people (control group, age <90 years). No subject had a history of cardiovascular disease. In longevity group,100 people were from the Longevity and Health of Aging Population (LHAP) study conducted in Bama County, Guangxi, China, in 2008 and had an average age of 96.9 ± 4.17 years. Peripheral blood was collected from them to isolate Genomic DNA for WES. The other 5007 cases were from the Chinese elderly health longevity factors (CLHLS) survey and had an average age of 97.1 ± 5.08 years old. The control group was drawn entirely from the CLHLS survey and had an average age of 72.0±13.72 years.

Phenotypic information, including age, gender and body mass index (BMI) were recorded. Laboratory test data including total cholesterol (TC), triglyceride (TG), high-density lipoprotein (HDL-c), and low-density lipoprotein (LDL-c) were also recorded. For detailed information, see Figure 1.

WES and quality controls

After DNA extraction, 2 μg of genomic DNA was taken and fragmented by sonication. The main fragment, measuring 200 bp, was recovered by isolating the section of the gel corresponding to 150-250 bp. The DNA fragment was end repaired using the Klenow fragment. T-ligase was used to add A to the 5' end of the fragment, and then the linker was ligated. After ligation of the sequencing linker and purification of the product, the genes were amplified by PCR. The amplified product was purified into a standby library. DNA from the library was hybridized with the probe in the Whole Exome Capture Kit (Agilent,39M, California, USA). Hybridization product was eluted and recovered for PCR amplification. The recovered product was the final library. Agarose gel electrophoresis was used to confirm the sample. The library was quality controlled by qPCR, and the validated library was sequenced using the Illumina HiSeq 2500 V4 sequencing platform at a setting of 125 PE. The average sequencing multiplier was no less than 50× per sample. Sanger sequencing was used for genotyping in the case-control study. For genotyping quality control, all carriers of associated alleles as well as 10% of cases and 10% of controls who carried only nonrisk alleles were regenotyped by Sanger sequencing.

GWAS genotype and quality controls

After DNA extraction, 14864 subjects were genotyped using Affymetrix Chips, which were created by strategically selecting optimized tag single-nucleotide polymorphism (SNP) content from all three HapMap phases and the 1000 Genomes Project (1KGP). A total of 13776 samples were passed through quality control, including 5313 long-lived people and 8463 middle-aged controls. A total of 5.6 million SNP sites were detected per sample (32,448 probes, 5215 probes at the same site).

The quality control for the experimental process consisted mainly of dish QC (DQC) and call rate (CR). DQC is applied to control the quality of a batch of samples (DQC ≥ 0.82) and eliminate pollution and insufficient signal intensity. CR is used to quality control typing results and select the sample with the highest CR (≥ 0.97) for later analysis. In the pretest phase, some batches of experimental data fluctuated. To ensure the accuracy of typing, we added a positive control and tested its typing accuracy to judge the classification accuracy of the same batch of samples. After DNA extraction, 14864 people were genotyped using Affymetrix chips, which were created by strategically selecting optimized tag SNP content from all three HapMap phases and 1KGP. The genomic inflation factors (λ) in the datasets were 1.027, suggesting the effects of population stratification on genetic analysis are well controlled, which means that the population structure has very little effect on the results.The association test statistics conformed to the underlying null distribution and would not require further adjustment for genomic control.

Discovery-evaluation strategy

All the single-nucleotide variant (SNV) allele data from exon sequencing of 100 long-lived individuals (longevity group 1, mean age (96.9±4.17) years old) were read and compared with the allele frequency distribution in the 1KGP Southern Han Chinese (CHS) population database. P<0.05 was used as the significance level, and mutations with an allele frequency of 0 in the CHS population were removed. A total of 395 long-lived individuals from the CLHLS (longevity group 2, mean age (102.3±3.18) years old) were selected for the GWAS. $P<10^{-4}$ was used as the significance level, and mutations with an allele frequency of 0 in the CHS population were removed.

We combined the variants shared in the above steps and compared them with the 1KGP III Chinese population (control group 1, including the CHS, CHB, and CDX populations; 301 people in all, age <60 years old), selecting the modest a priori discovery threshold of $P<10^{-4}$ [33–35]. The common variations were replicated and verified in 5107 long-lived individuals (longevity group) and 8469 non-long-lived elderly people (control group). Following the usual practice, we applied a nominal significance level of $P<0.05$ here as well as in phenotypic stratification analysis.

Bioinformatics functional analysis

SIFT and PolyPhen 2 software were used to jointly predict the effects of the variants on the functions of their genes. SFmap software was used to predict the effects of mutations on the splicing of genes during transcription. SWISS-MODEL software was used to predict the structures of the mutant protein sequences. We analyzed our experimental results in the functional context of gene-gene networks from multiple organisms in IMP. In the functional gene networks constructed with IMP, genes connected by an edge are predicted to participate in similar biological processes. Kyoto Encyclopedia of Genes and Genomes (KEGG) signaling pathway analysis revealed that TFPI rs7586970 and ADAMTS7 rs3825807 are associated with healthy longevity through their effects on lipid metabolism balance.

Meta-analysis of the effect of TFPI rs7586970 T/C on plasma TFPI concentration

Tissue factor (TF) is one of the major initiators of coagulation, and increased plasma levels have been found in various CVDs. TF activity is, however, regulated by TFPI, and alteration in levels of TF and/or TFPI may thus relate to thrombogenesis and atherogenesis. Previous studies have shown that the TFPI rs7586970 T/C mutation affects plasma total TFPI concentration. Decreased TFPI concentration will increase the risk of venous thromboembolism (VTE) and myocardial infarction (MI) [10, 41]. This study systematically evaluated the effects of the TFPI rs7586970 T/C polymorphism on plasma TFPI concentrations in six previous studies.

SUPPLEMENTARY RESULTS

TFPI and ADAMTS7

There were interactions between the TFPI gene and the SPARC gene (relationship confidence weight 0.482, chemical and genetic perturbations 0.1687), the SPARC gene and the PDGFRB gene (relationship confidence weight 0.374, chemical and genetic perturbations 0.1687), and the PDGFRB gene and the ADAMTS7 gene (weight 7074, chemical and genetic perturbations 0.363).

TFPI and *APOE*

The TFPI gene interacts indirectly with APOE via the LDLR gene. Most free TFPI in the human body binds to lipoprotein. TFPI can bind to some cell surface receptors, such as LDL receptor (LDLR)-related proteins (known as Low-Density Lipoprotein Receptor Associated Protein, LRP), through its K3 fragment and carboxy-terminal structure to become a lipoprotein-associated coagulation inhibitor (LACI) that alters the distribution of very low-density lipoprotein (VLDL) and degrades it. Consequently, it reduces the levels of plasma triglycerides and LDL. The LRP1 protein is a member of the LDLR protein family. There is a functional interaction between the APOE gene and the LRP1 gene that is predicted to participate in similar biological processes in a functional network (relationship confidence weight 0.732, protein-protein interactions weight 0.7645).

TFPI interacts indirectly with APOE via DAB2 and LRP1. According to the IMP system search results, the TFPI gene interacts with the DAB2 gene (relationship confidence weight 0.496, chemical and genetic perturbations 0.1687). DAB2 interacts with the LRP1 gene (relationship confidence weight 0.538, physical and genetic interactions 0.3652). LRP1 interacts with the APOE gene (relationship confidence weight 0.793, physical and genetic interactions 0.3652).

APOE and *ADAMTS7*

ADAMTS7 interacts indirectly with the APOE gene via PDGFRB. PDGFRB interacts with ADAMTS7 (relationship confidence weight 0.374, chemical and genetic perturbations 0.1687). PDGFRB interacts with APOE (relationship confidence weight 0.419, chemical and genetic perturbations 0.1687) (Supplementary Figure 4).
