# Supplementary material for: Identification of cardiovascular health gene variants related to longevity in a Chinese population
Source: Aging (Albany NY). 2020 Sep 7;12(17):16775–802. doi: 10.18632/aging.103396 (PMC7521493; doi:10.18632/aging.103396)
Supplement: Supplementary Figures [file aging-12-103396-s009..pdf]

SUPPLEMENTARY FIGURES

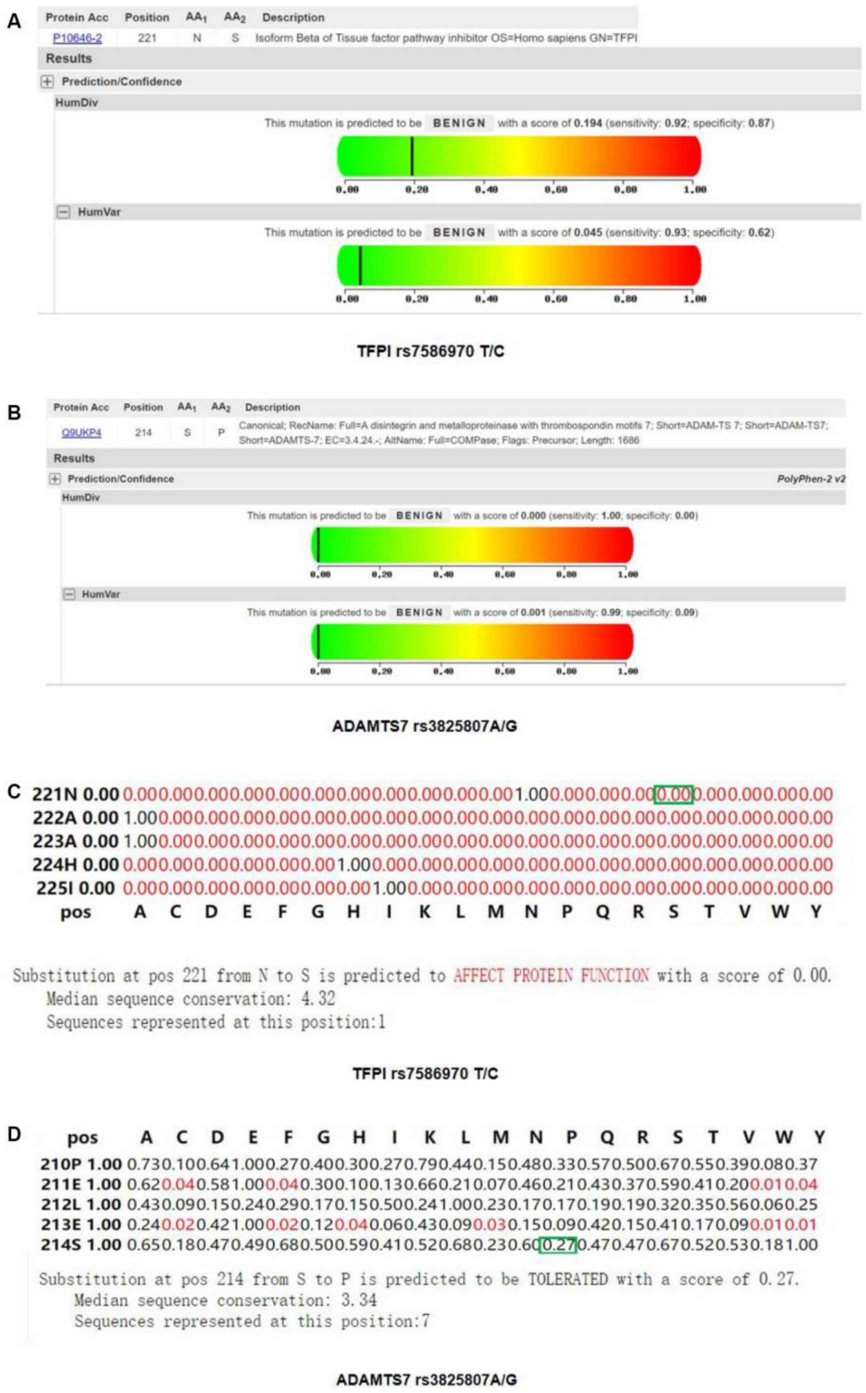

Supplementary Figure 1. Predicted results of effect of variants on protein expression. (**A** and **B**) Polyphen2 analysis results show that the effects of rs7586970 and rs3825807 variations on TFPI and ADAMTS7 are benign. (**C** and **D**) SIFT software analysis results show that rs7586970 variation affects function of TFPI protein,but rs3825807 variation did not affect the function of the ADAMTS7 protein.

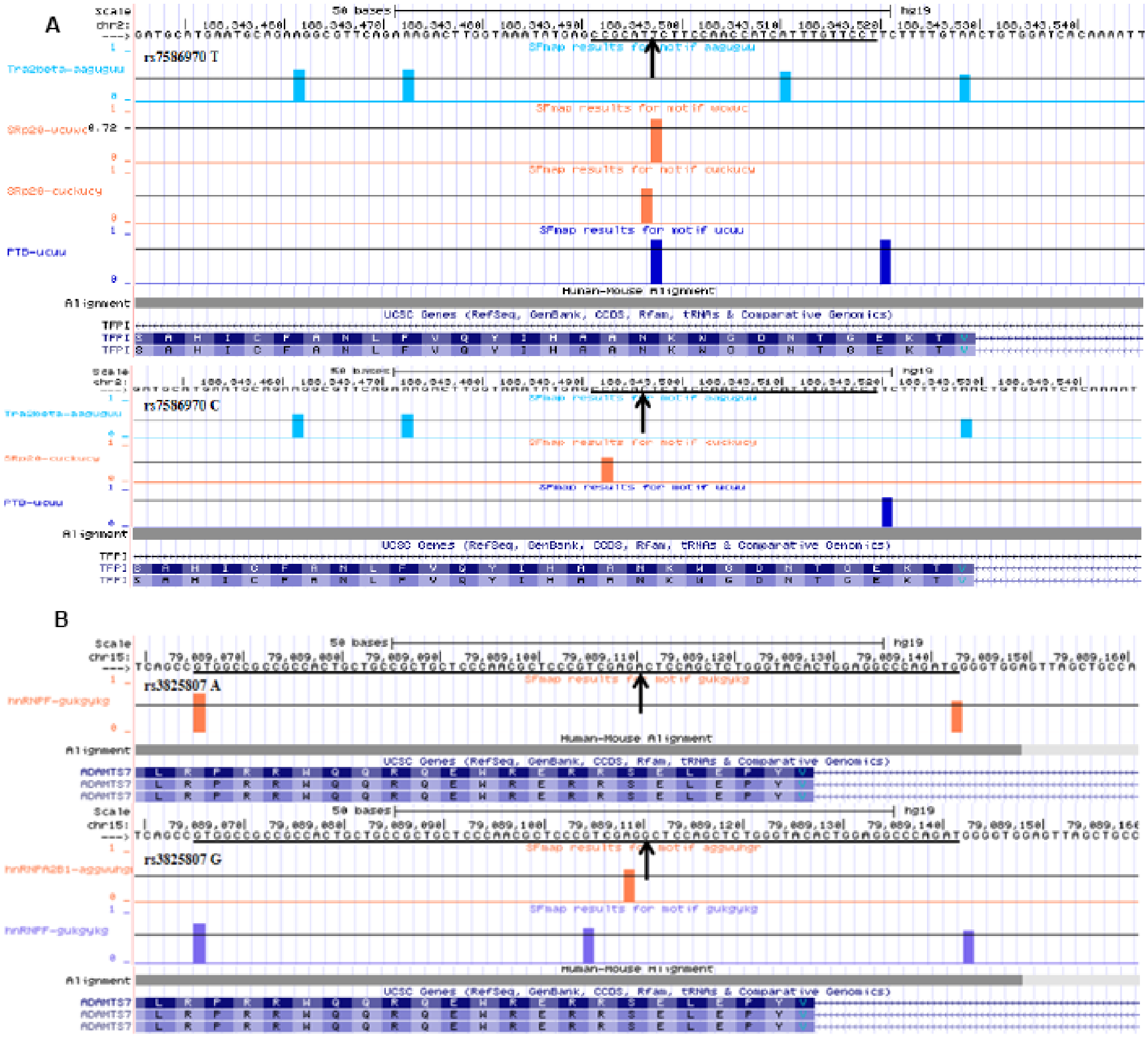

Supplementary Figure 2. Prediction of splicing factors and motif analysis of TFPI rs7586970 and ADAMTS7 rs3825807. (**A**) Function annotation of TFPI rs7586970 T/C. The TFPI rs7586970-C variant disrupts the binding site of Tra2β (Transformer-2 protein homolog beta) and PTB (polypyrimidine tract binding protein polypyrimidine tract binding protein) and changes the splice position of the splicing factor in SRp20. (**B**) Function annotation of ADAMTS7 rs3825807 A/G. The ADAMTS7 rs3825807-G variant changes the splicing position of hnRNPF (Heterogeneous nuclear ribonucleoproteins F) and hnRNPA2B1 (Heterogeneous nuclear ribonucleoproteins A2B1).

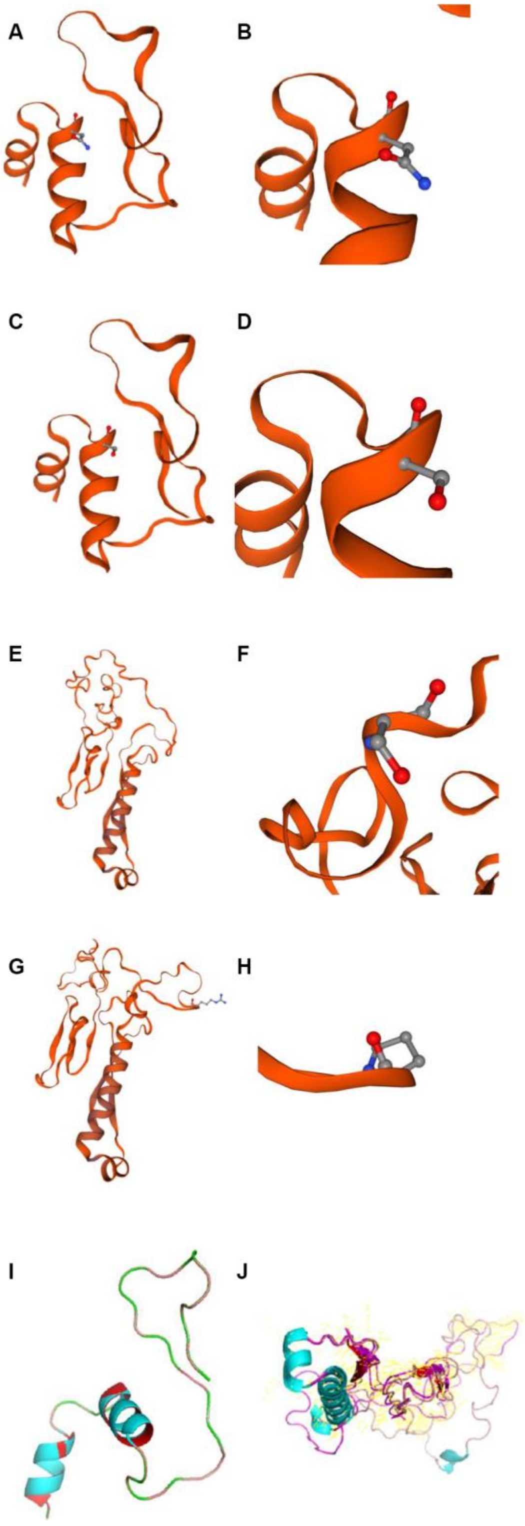

Supplementary Figure 3. Spatial Structural Prediction of Mutant Protein. (**A**) Spatial structural of TFPI rs7586970 T. (**B**) Amino acid in TFPI rs7586970 T. (**C**) Spatial structural of TFPI rs7586970 C. (**D**) Amino acid in TFPI rs7586970 C. (**E**) Spatial structural of ADAMTS7 rs3825807 A. (**F**) Amino acid in ADAMTS7 rs3825807 A. (**G**) Spatial structural of ADAMTS7 rs3825807 G. (**H**) Amino acid in ADAMTS7 rs3825807 G. (**I**) Comparing structure of TFPI rs7586970 T and TFPI rs7586970 C. (**J**) Comparing structure of ADAMTS7 rs3825807 A and ADAMTS7 rs3825807 G.

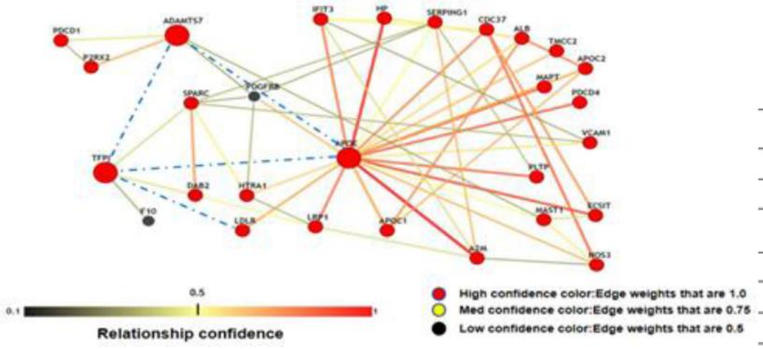

Gene	DAB2	LRP1	SPARC	PDGFR β
Weight	RC	RC	RC	RC
TFPI	0.496		0.482	-
ADAMTS7	-	-	-	0.374
APOE	-	0.732	-	0.419
PDGFR β	-	-	0.374	-
LRP1	0.538	-	-	-

Supplementary Figure 4. IMP interactive pathway analysis of TFPI and ADAMTS7 in longevity. Relationship confidence (RC): Edge weights in the above network, which reflect the probability that the two connected genes are functionally related. Functional gene network : genes connected by an edge in a functional network are predicted to participate in similar biological process.

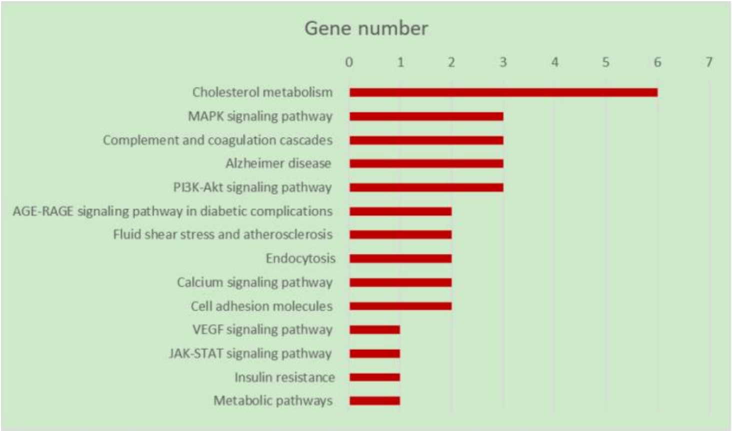

Supplementary Figure 5. KEGG pathway enrichment.

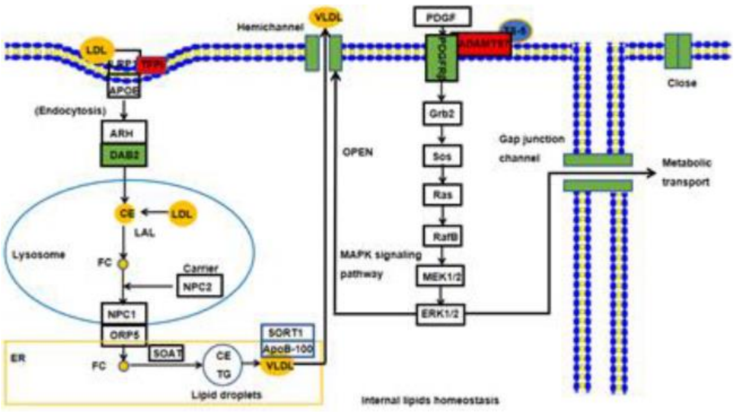

Supplementary Figure 6. Mechanism or interactive pathway of TFPI, ADAMTS7 and APOE in lipid metabolism homeostasis in longevity. Cholesterol metabolism: LRP1: Low Density Lipoprotein Receptor (LDLR) Associated Protein1. APOE: Apolipoprotein E. ARH: LDLRAP1, low density lipoprotein receptor adapter protein 1. DAB2: disabled homolog 2. LAL: LIPA, lysosomal acid lipase/cholesteryl ester hydrolase. NPC1: Niemann-Pick C1 protein. NPC2: Niemann-Pick C2 protein. ORP5: oxysterol-binding protein-related protein 5. SOAT: sterol-O-acyltransferase. SORT1: sortilin. ApoB: apolipoprotein. B. LDL: Low Density Lipoprotein. VLDL: Very low Density Lipoprotein. CE: Cholesterol ester. FC: Cholesterol. TG: Triacylglycerol.

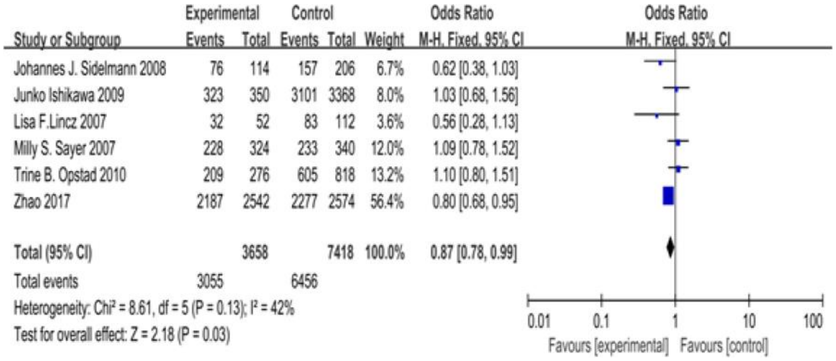

Supplementary Figure 7. Random effects meta-analysis results for TFPI rs7586970 and TFPI plasma levels under a codominant genetic model. Carriers of the rs7586970 T allele have a significantly reduced risk of venous thrombosis. Meta-analysis results showed that among 1829 venous thrombosis patients and normal controls, plasma TFPI concentration was significantly reduced in carriers of the rs7586970 T allele (p=0.03, OR=0.87).
