# Supplementary material for: Identification of cardiovascular health gene variants related to longevity in a Chinese population
Source: Aging (Albany NY). 2020 Sep 7;12(17):16775–802. doi: 10.18632/aging.103396 (PMC7521493; doi:10.18632/aging.103396)
Supplement: Supplementary Table 1 [file aging-12-103396-s002..docx]

**Supplementary Table 1. Candidate Variants associated longevity.**

|  |  | Major allele | Minor allele |  |
| --- | --- | --- | --- | --- |
| Variants | Chr. | Longevity/control | Longevity/control | p |
| rs17114036 | 1 | 784/587 | 6/15 | 0.009 |
| rs17114046 | 1 | 787/589 | 3/13 | 0.002 |
| rs7586970 | 2 | 753/514 | 37/88 | 1.34E-10 |
| rs11684202 | 2 | 747/475 | 43/127 | 9.88E-19 |
| rs6725887 | 2 | 788/590 | 2/12 | 1.27E-03 |
| rs840616 | 2 | 754/517 | 36/85 | 3.52E-10 |
| rs10933436 | 2 | 661/305 | 129/297 | 5.26E-40 |
| rs11920719 | 3 | 785/583 | 5/19 | 3.40E-04 |
| rs17589290 | 4 | 765/538 | 27/64 | 6.41E-08 |
| rs1395821 | 4 | 707/383 | 83/219 | 4.00E-31 |
| rs10026364 | 4 | 788/585 | 2/17 | 4.20E-05 |
| rs11748327 | 5 | 764/523 | 26/79 | 5.92E-12 |
| rs17577085 | 5 | 779/576 | 11/26 | 7.71E-04 |
| rs13161895 | 5 | 733/432 | 57/170 | 7.07E-26 |
| rs3798220 | 6 | 769/543 | 21/59 | 1.41E-08 |
| rs675026 | 6 | 103/59 | 97/543 | 4.26E-37 |
| rs17609940 | 6 | 789/591 | 1/11 | 6.73E-04 |
| rs12190287 | 6 | 691/348 | 99/234 | 3.19E-32 |
| rs365302 | 6 | 721/427 | 69/175 | 4.79E-23 |
| rs7808424 | 7 | 682/358 | 108/224 | 2.75E-26 |
| rs10953541 | 7 | 751/490 | 39/112 | 4.52E-16 |
| rs13232179 | 7 | 689/323 | 101/279 | 4.50E-44 |
| rs4875320 | 8 | 766/506 | 24/96 | 1.87E-17 |
| rs1333049 | 9 | 723/289 | 67/313 | 7.40E-73 |
| rs7865618 | 9 | 768/546 | 22/56 | 1.62E-07 |
| rs4743150 | 9 | 759/547 | 31/55 | 6.30E-05 |
| rs514659 | 9 | 700/377 | 90/225 | 1.71E-30 |
| rs3739998 | 10 | 762/487 | 28/115 | 2.74E-21 |
| rs2185724 | 10 | 717/358 | 73/244 | 2.86E-43 |
| rs2505083 | 10 | 753/476 | 37/126 | 9.67E-21 |
| rs1746048 | 10 | 715/414 | 75/188 | 1.03E-24 |
| rs12413409 | 10 | 714/438 | 76/164 | 6.52E-18 |
| rs1412444 | 10 | 716/406 | 74/196 | 2.20E-27 |
| rs12269901 | 11 | 734/444 | 56/158 | 9.50E-23 |
| rs10895547 | 11 | 712/357 | 78/245 | 1.63E-41 |
| rs964184 | 11 | 755/469 | 35/133 | 1.22E-23 |
| rs4937126 | 11 | 685/327 | 105/275 | 3.58E-41 |
| rs3184504 | 12 | 100/3 | 100/599 | 1.90E-73 |
| rs10861032 | 12 | 666/312 | 124/290 | 2.16E-39 |
| rs9546711 | 13 | 692/349 | 98/253 | 1.90E-36 |
| rs2895811 | 14 | 731/455 | 59/147 | 1.11E-18 |
| rs12595292 | 15 | 724/442 | 66/160 | 6.60E-20 |
| rs3825807 | 15 | 762/510 | 28/92 | 1.07E-14 |
| rs937254 | 15 | 660/342 | 130/260 | 3.68E-28 |
| rs1994016 | 15 | 774/543 | 16/59 | 1.95E-10 |
| rs7203193 | 16 | 688/314 | 102/288 | 7.27E-47 |
| rs16965039 | 16 | 783/584 | 7/18 | 0.003 |
| rs12936587 | 17 | 764/536 | 26/66 | 1.14E-08 |
| rs1231206 | 17 | 734/450 | 56/152 | 4.70E-21 |
| rs216172 | 17 | 734/450 | 56/152 | 4.70E-21 |
| rs46522 | 17 | 714/409 | 76/193 | 8.23E-26 |
| rs1122608 | 19 | 772/558 | 18/44 | 7.00E-06 |
| rs11671653 | 19 | 772/562 | 18/40 | 5.40E-05 |
| rs4804155 | 19 | 737/464 | 53/138 | 3.02E-18 |
| rs1475591 | 21 | 774/544 | 16/58 | 3.63E-10 |
| rs1735151 | 21 | 713/396 | 77/206 | 2.61E-29 |
