# Supplementary material for: Identification of cardiovascular health gene variants related to longevity in a Chinese population
Source: Aging (Albany NY). 2020 Sep 7;12(17):16775–802. doi: 10.18632/aging.103396 (PMC7521493; doi:10.18632/aging.103396)
Supplement: Supplementary Tables 2, 3 and 4 [file aging-12-103396-s007..pdf]

SUPPLEMENTARY TABLES

Supplementary Table 2. Variants associated longevity.

SNP	Chr.	Gene	Major homo Longevity/Control	Minor homo Longevity/Control	Hetero Longevity/Control	P	Major homo/Minor homo			Major homo/Hetero			Minor homo/Hetero		
							P	OR	95%CI	P	OR	95%CI	P	OR	95%CI
rs7586970	2	TFPI	3801/6256	44/141	1093/1867	4.09E-04	9.60E-05	1.947	1.385-2.738	0.391	1.038	0.953-1.130	3.07E-04	0.533	0.377-0.754
rs3825807	15	ADAMTS7	929/1918	18/47	242/611	0.050	0.401	1.265	0.73-2.190	0.019	1.223	1.034-1.447	0.907	0.967	0.551-1.698
SNP	Chr.	Gene	Centenarians/ Nonagenarians	Centenarians/ Nonagenarians	Centenarians/ Nonagenarians	P	P	OR	95%CI	P	OR	95%CI	P	OR	95%CI
rs7586970	2	TFPI	1466/2335	10/34	366/727	0.001	0.032	2.135	1.051-4.334	0.002	1.247	1.082-1.437	0.137	0.584	0.285-1.196
rs3825807	15	ADAMTS7	503/426	5/13	103/139	7.51E-04	0.027	3.07	1.086-8.681	0.001	1.593	1.198-2.120	0.219	0.519	0.179-1.502
SNP	Chr.	Gene	Centenarians/ Control	Centenarians/ Control	Centenarians/ Control	P	P	OR	95%CI	P	OR	95%CI	P	OR	95%CI
rs7586970	2	TFPI	1466/6256	10/141	366/1867	2.0E-05	1.16E-04	3.304	1.735-6.291	0.005	1.195	1.054-1.355	0.001	0.362	0.189-0.694
rs3825807	15	ADAMTS7	503/1918	5/47	103/611	1.61E-04	0.049	2.465	0.975-6.230	1.59E-04	1.556	1.235-1.959	0.336	0.631	0.245-1.624
SNP	Chr.	Gene	Nonagenarians/Control	Nonagenarians /Control	Nonagenarians /Control	P	P	OR	95%CI	P	OR	95%CI	P	OR	95%CI
rs7586970	2	TFPI	2335/6256	34/141	727/1867	0.045	0.022	1.548	1.061-2.258	0.397	0.959	0.869-1.057	0.014	0.619	0.422-0.909
rs3825807	15	ADAMTS7	426/1918	13/47	139/611	0.777	0.489	0.803	.431-1.497	0.825	0.976	0.79-1.207	0.550	1.216	0.64-2.309

Supplementary Table 3. Comparative analysis baseline information about phenotype information between longevity and control.

	Centenarians	Nonagenarians	Longevity	Control	P (Cen. vs Non.)	P (Long. vs Con.)	P (Cen. vs Con.)	P (Non. vs Con)
Age	102.3±2.19	94.0±2.76	98.2±4.86	69.7±13.97	0.000	0.000	0.000	0.000
Gender								
Male	355	339	694	1086				
Female	510	505	1015	1480	0.713	0.265	0.509	0.270
BMI	24.9±9.546	23.2±2.557	24.0±7.021	22.2±2.542	0.265	0.001	0.060	0.001
FBG	5.1±2.24	4.9±2.08	5.0±2.17	5.0±2.16	0.021	0.047	0.004	0.827
TC	4.2±1.177	4.1±1.16	4.1±1.17	4.1±1.18	0.426	0.983	0.627	0.642
TG	1.27±0.99	1.2±0.93	1.3±0.96	1.2±1.00	0.569	0.011	0.019	0.099
LDL	2.4±0.85	2.4±0.87	2.4±0.86	2.42±0.86	0.728	0.428	0.666	0.408
HDL	1.28±0.374	1.23±0.347	1.26±0.361	1.26±0.344	0.038	0.225	0.766	0.025

Cen. stands for Centenarians.
Non. stands for Nonagenarians.
Long. stands for Longevity.
Con. stands for Control.

Supplementary Table 4. Association of TFPI rs7586970 and ADAMTS7 rs3825807 with phenotype.

rs7586970	Centenarians			Nonagenarians			Longevity			Control			P(Cen./Non.)	P(Cen./Con.)	P(Non./Con.)	P(Long./Con.)
	TT	CC	TC	TT	CC	TC	TT	CC	TC	TT	CC	TC				
BMI																
BMI (+)	446	4	96	365	10	135	811	14	231	1208	24	342	4.290E-04	0.043	0.065	0.918
BMI (-)	217	2	49	190	7	61	407	9	110	507	23	158	0.065	0.014	0.878	0.116
FBG																
FBG (+)	412	5	80	377	12	131	789	17	211	1034	26	287	3.440E-04	0.020	0.135	0.930
FBG (-)	48	0	5	26	0	9	74	0	14	120	2	28	0.085	0.302	0.437	0.855
TC																
TC (+)	416	5	76	375	12	133	791	17	209	1054	27	298	5.200E-05	0.004	0.130	0.791
TC (-)	44	0	9	28	0	7	72	0	16	100	1	17	0.898	0.848	0.471	0.821
TG																
TG (+)	376	4	69	324	12	125	700	16	194	951	23	259	1.000E-05	0.009	0.017	0.824
TG (-)	84	1	16	79	0	15	163	1	31	203	5	56	0.792	0.491	0.423	0.243
LDL																
LDL (+)	394	4	68	364	12	125	758	16	193	992	26	274	3.800E-05	0.002	0.162	0.718
LDL (-)	67	1	17	39	0	15	106	1	32	163	2	41	0.574	1.000	0.394	0.785
HDL																
HDL (+)	443	5	84	385	12	135	828	17	219	1107	27	302	0.95E-04	0.013	0.080	0.938
HDL (-)	17	0	1	17	0	5	34	0	6	47	1	13	0.537	0.435	0.904	0.834

rs3825807	Centenarians			Nonagenarians			Longevity			Control			P (Cen./Non.)	P (Cen./Con.)	P (Non./Con.)	P (Long./Con.)
	AA	GG	AG	AA	GG	AG	AA	GG	AG	AA	GG	AG				
BMI																
BMI (+)	241	1	37	155	7	28	396	8	65	413	11	113	0.016	0.001	0.091	0.010
BMI (-)	137	2	20	67	4	31	204	6	51	178	5	57	3.100E-04	0.011	0.239	0.518
FBG																
FBG (+)	345	2	51	180	10	49	525	12	100	519	11	143	8.700E-05	4.760E-04	0.078	0.035
FBG (-)	33	1	4	25	0	4	58	1	8	48	1	22	0.891	0.028	0.207	0.010
TC																
TC (+)	338	3	50	196	9	50	534	12	100	508	9	154	0.002	1.520E-04	0.063	0.002
TC (-)	40	0	5	9	1	3	49	1	8	59	3	11	0.069	0.694	0.381	0.859
TG																
TG (+)	310	2	44	176	10	45	486	12	89	465	10	140	2.720E-04	8.900E-05	0.049	0.003
TG (-)	67	1	11	29	0	8	96	1	19	101	2	25	0.524	0.633	0.924	0.712
LDL																
LDL (+)	316	3	49	183	9	48	499	12	97	474	8	146	0.002	4.770E-04	0.044	0.004
LDL (-)	62	0	6	22	1	5	84	1	11	94	4	19	0.124	0.264	0.909	0.347
HDL																
HDL (+)	252	2	36	143	8	40	395	10	76	383	6	120	8.63E-04	4.56E-04	0.035	0.006
HDL (-)	14	1	1	10	0	0	24	1	1	30	1	7	1	0.460	0.606	0.181

Cen. stands for Centenarians.
Non. stands for Nonagenarians.
Long. stands for longevity.
Con. stands for control.
