# Supplementary material for: Identification of cardiovascular health gene variants related to longevity in a Chinese population
Source: Aging (Albany NY). 2020 Sep 7;12(17):16775–802. doi: 10.18632/aging.103396 (PMC7521493; doi:10.18632/aging.103396)
Supplement: Supplementary Table 5 [file aging-12-103396-s001..docx]

**Supplementary Table 5. Stratification analysis of metabolic phenotype with polymorphism of TFPI rs7586970 or ADAMTS7 rs3825807.**

|  | rs7586970 |  |  |  |  |  | rs7586970 |  |  |  |  |  | rs3825807 |  |  |  |  |  | rs3825807 |  |  |  |  |  |
| --- | --- | --- | --- | --- | --- | --- | --- | --- | --- | --- | --- | --- | --- | --- | --- | --- | --- | --- | --- | --- | --- | --- | --- | --- |
| Centenarians/Nonagenarians | T | C | p | OR | 95%CI |  | TT | TC+CC | p | OR | 95%CI |  | A | G | p | OR | 95%CI |  | AA | AG+GG | p | OR | 95%CI |  |
| Lipids（-）+FBG（-）+BMI（-） |  |  |  |  |  |  |  |  |  |  |  |  |  |  |  |  |  |  |  |  |  |  |  |  |
| Centenarians | 375 | 39 |  |  |  |  | 170 | 37 |  |  |  |  | 324 | 24 |  |  |  |  | 150 | 24 |  |  |  |  |
| Nonagenarians | 428 | 78 | 0.007 | 1.752 | 1.165 | 2.637 | 179 | 74 | 0.005 | 1.899 | 1.215 | 2.97 | 211 | 35 | 0.003 | 2.239 | 1.295 | 3.872 | 94 | 29 | 0.030 | 1.928 | 1.059 | 3.51 |
| Lipids（-）+FBG（-）+BMI（+） |  |  |  |  |  |  |  |  |  |  |  |  |  |  |  |  |  |  |  |  |  |  |  |  |
| Centenarians | 179 | 13 |  |  |  |  | 84 | 12 |  |  |  |  | 169 | 15 |  |  |  |  | 78 | 14 |  |  |  |  |
| Nonagenarians | 164 | 34 | 0.002 | 2.855 | 1.456 | 5.597 | 70 | 29 | 0.004 | 2.9 | 1.378 | 6.101 | 104 | 22 | 0.013 | 2.383 | 1.183 | 4.801 | 44 | 19 | 0.026 | 2.406 | 1.1 | 5.264 |
| Lipids（-）+FBG（+）+BMI（-） |  |  |  |  |  |  |  |  |  |  |  |  |  |  |  |  |  |  |  |  |  |  |  |  |
| Centenarians | 41 | 3 |  |  |  |  | 19 | 3 |  |  |  |  | 22 | 4 |  |  |  |  | 10 | 3 |  |  |  |  |
| Nonagenarians | 28 | 4 | 0.657 | 1.952 | 0.405 | 9.405 | 12 | 4 | 0.640 | 2.111 | 0.4 | 11.128 | 24 | 2 | 0.664 | 0.458 | 0.076 | 2.754 | 11 | 2 | 1.000 | 0.606 | 0.083 | 4.405 |
| Lipids（+）+FBG（-）+BMI（-） |  |  |  |  |  |  |  |  |  |  |  |  |  |  |  |  |  |  |  |  |  |  |  |  |
| Centenarians | 211 | 17 |  |  |  |  | 98 | 16 |  |  |  |  | 152 | 8 |  |  |  |  | 72 | 8 |  |  |  |  |
| Nonagenarians | 150 | 28 | 0.008 | 2.317 | 1.224 | 4.385 | 63 | 26 | 0.008 | 2.528 | 1.257 | 5.083 | 50 | 2 | 1.000 | 0.76 | 0.156 | 3.697 | 24 | 2 | 1.000 | 0.75 | 0.149 | 3.778 |
| Lipids（-）+FBG（+）+BMI（+） |  |  |  |  |  |  |  |  |  |  |  |  |  |  |  |  |  |  |  |  |  |  |  |  |
| Centenarians | 23 | 1 |  |  |  |  | 11 | 1 |  |  |  |  | 22 | 0 |  |  |  |  | 11 | 0 |  |  |  |  |
| Nonagenarians | 10 | 2 | 0.522 | 4.6 | 0.373 | 56.752 | 4 | 2 | 0.502 | 5.5 | 0.385 | 78.573 | 6 | 0 | 0.444 | 3.286 | 0.181 | 59.599 | 3 | 0 | 0.490 | 3 | 0.15 | 59.89 |
| Lipids（+）+FBG（-）+BMI（+） |  |  |  |  |  |  |  |  |  |  |  |  |  |  |  |  |  |  |  |  |  |  |  |  |
| Centenarians | 103 | 21 |  |  |  |  | 42 | 20 |  |  |  |  | 86 | 8 |  |  |  |  | 40 | 7 |  |  |  |  |
| Nonagenarians | 125 | 15 | 0.142 | 0.589 | 0.289 | 1.2 | 56 | 14 | 0.108 | 0.525 | 0.238 | 1.159 | 28 | 10 | 0.007 | 3.839 | 1.381 | 10.677 | 10 | 9 | 0.014 | 5.143 | 1.539 | 17.187 |
| Lipids（+）+FBG（+）+BMI（-） |  |  |  |  |  |  |  |  |  |  |  |  |  |  |  |  |  |  |  |  |  |  |  |  |
| Centenarians | 19 | 1 |  |  |  |  | 9 | 1 |  |  |  |  | 16 | 0 |  |  |  |  | 8 | 0 |  |  |  |  |
| Nonagenarians | 9 | 1 | 1.000 | 2.111 | 0.118 | 37.722 | 4 | 1 | 1.000 | 2.25 | 0.111 | 45.723 | 14 | 0 | 1.000 | 1.133 | 0.065 | 19.739 | 7 | 0 | 1.000 | 1.125 | 0.06 | 21.087 |
| Lipids（+）+FBG（+）+BMI（+） |  |  |  |  |  |  |  |  |  |  |  |  |  |  |  |  |  |  |  |  |  |  |  |  |
| Centenarians | 16 | 0 |  |  |  |  | 8 | 0 |  |  |  |  | 7 | 1 |  |  |  |  | 3 | 1 |  |  |  |  |
| Nonagenarians | 14 | 2 | 0.596 | 3.4 | 0.319 | 36.272 | 6 | 2 | 0.576 | 3.857 | 0.326 | 45.57 | 10 | 2 | 1.000 | 1.4 | 0.105 | 18.615 | 4 | 2 | 1.000 | 1.500 | .089 | 25.392 |
| Centenarians/Control |  |  |  |  |  |  |  |  |  |  |  |  |  |  |  |  |  |  |  |  |  |  |  |  |
| Lipids（-）+FBG（-）+BMI（-） |  |  |  |  |  |  |  |  |  |  |  |  |  |  |  |  |  |  |  |  |  |  |  |  |
| Centenarians | 375 | 39 |  |  |  |  | 170 | 37 |  |  |  |  | 324 | 24 |  |  |  |  | 150 | 24 |  |  |  |  |
| Control | 1197 | 159 | 0.193 | 1.277 | 0.883 | 1.847 | 528 | 150 | 0.190 | 1.305 | 0.876 | 1.946 | 583 | 81 | 0.009 | 1.876 | 1.166 | 3.017 | 256 | 76 | 0.015 | 1.855 | 1.124 | 3.063 |
| Lipids（-）+FBG（-）+BMI（+） |  |  |  |  |  |  |  |  |  |  |  |  |  |  |  |  |  |  |  |  |  |  |  |  |
| Centenarians | 179 | 13 |  |  |  |  | 84 | 12 |  |  |  |  | 169 | 15 |  |  |  |  | 78 | 14 |  |  |  |  |
| Control | 353 | 57 | 0.011 | 2.223 | 1.186 | 4.169 | 156 | 49 | 0.022 | 2.199 | 1.109 | 4.361 | 241 | 39 | 0.058 | 1.823 | 0.974 | 3.413 | 102 | 38 | 0.033 | 2.076 | 1.052 | 4.097 |
| Lipids（-）+FBG（+）+BMI（-） |  |  |  |  |  |  |  |  |  |  |  |  |  |  |  |  |  |  |  |  |  |  |  |  |
| Centenarians | 41 | 3 |  |  |  |  | 19 | 3 |  |  |  |  | 22 | 4 |  |  |  |  | 10 | 3 |  |  |  |  |
| Control | 88 | 10 | 0.740 | 1.553 | 0.406 | 5.945 | 40 | 9 | 0.881 | 1.425 | 0.346 | 5.873 | 51 | 11 | 1.000 | 1.186 | 0.34 | 4.136 | 20 | 11 | 0.652 | 1.833 | 0.415 | 8.096 |
| Lipids（+）+FBG（-）+BMI（-） |  |  |  |  |  |  |  |  |  |  |  |  |  |  |  |  |  |  |  |  |  |  |  |  |
| Centenarians | 211 | 17 |  |  |  |  | 98 | 16 |  |  |  |  | 152 | 8 |  |  |  |  | 72 | 8 |  |  |  |  |
| Control | 513 | 69 | 0.068 | 1.669 | 0.959 | 2.906 | 227 | 64 | 0.070 | 1.727 | 0.951 | 3.137 | 216 | 24 | 0.071 | 2.111 | 0.924 | 4.825 | 97 | 23 | 0.079 | 2.134 | 0.903 | 5.045 |
| Lipids（-）+FBG（+）+BMI（+） |  |  |  |  |  |  |  |  |  |  |  |  |  |  |  |  |  |  |  |  |  |  |  |  |
| Centenarians | 23 | 1 |  |  |  |  | 11 | 1 |  |  |  |  | 22 | 0 |  |  |  |  | 11 | 0 |  |  |  |  |
| Control | 34 | 6 | 0.352 | 4.059 | 0.458 | 35.982 | 15 | 5 | 0.483 | 3.667 | 0.374 | 35.979 | 22 | 2 | 0.661 | 3 | 0.29 | 31.013 | 10 | 2 | 0.644 | 3.273 | 0.295 | 36.311 |
| Lipids（+）+FBG（-）+BMI（+） |  |  |  |  |  |  |  |  |  |  |  |  |  |  |  |  |  |  |  |  |  |  |  |  |
| Centenarians | 103 | 21 |  |  |  |  | 42 | 20 |  |  |  |  | 86 | 8 |  |  |  |  | 40 | 7 |  |  |  |  |
| Control | 247 | 47 | 0.810 | 0.933 | 0.531 | 1.64 | 103 | 44 | 0.739 | 0.897 | 0.474 | 1.699 | 116 | 16 | 0.385 | 1.483 | 0.607 | 3.623 | 53 | 13 | 0.510 | 1.402 | 0.512 | 3.834 |
| Lipids（+）+FBG（+）+BMI（-） |  |  |  |  |  |  |  |  |  |  |  |  |  |  |  |  |  |  |  |  |  |  |  |  |
| Centenarians | 19 | 1 |  |  |  |  | 9 | 1 |  |  |  |  | 16 | 0 |  |  |  |  | 8 | 0 |  |  |  |  |
| Control | 55 | 7 | 0.696 | 2.418 | 0.279 | 20.952 | 24 | 7 | 0.679 | 2.625 | 0.282 | 24.435 | 25 | 7 | 0.213 | 5.231 | 0.599 | 45.67 | 10 | 6 | 0.236 | 5.727 | 0.59 | 55.6 |
| Lipids（+）+FBG（+）+BMI（+） |  |  |  |  |  |  |  |  |  |  |  |  |  |  |  |  |  |  |  |  |  |  |  |  |
| Centenarians | 16 | 0 |  |  |  |  | 8 | 0 |  |  |  |  | 7 | 1 |  |  |  |  | 3 | 1 |  |  |  |  |
| Control | 79 | 7 | 0.979 | 1.7 | 0.199 | 14.503 | 36 | 7 | 0.897 | 1.946 | 0.215 | 17.611 | 16 | 4 | 1.000 | 1.75 | 0.164 | 18.617 | 6 | 4 | 1.000 | 2 | 0.15 | 26.734 |
| Nonagenarians/Control |  |  |  |  |  |  |  |  |  |  |  |  |  |  |  |  |  |  |  |  |  |  |  |  |
| Lipids（-）+FBG（-）+BMI（-） |  |  |  |  |  |  |  |  |  |  |  |  |  |  |  |  |  |  |  |  |  |  |  |  |
| Nonagenarians | 428 | 78 |  |  |  |  | 179 | 74 |  |  |  |  | 211 | 35 |  |  |  |  | 94 | 29 |  |  |  |  |
| Control | 1197 | 159 | 0.034 | 0.729 | 0.544 | 0.977 | 528 | 150 | 0.024 | 0.687 | 0.496 | 0.952 | 583 | 81 | 0.415 | 0.838 | 0.547 | 1.283 | 256 | 76 | 0.877 | 0.962 | 0.59 | 1.569 |
| Lipids（-）+FBG（-）+BMI（+） |  |  |  |  |  |  |  |  |  |  |  |  |  |  |  |  |  |  |  |  |  |  |  |  |
| Nonagenarians | 164 | 34 |  |  |  |  | 70 | 29 |  |  |  |  | 104 | 22 |  |  |  |  | 44 | 19 |  |  |  |  |
| Control | 353 | 57 | 0.290 | 0.779 | 0.49 | 1.238 | 156 | 49 | 0.313 | 0.758 | 0.442 | 1.3 | 241 | 39 | 0.357 | 0.765 | 0.432 | 1.354 | 102 | 38 | 0.658 | 0.863 | 0.448 | 1.66 |
| Lipids（-）+FBG（+）+BMI（-） |  |  |  |  |  |  |  |  |  |  |  |  |  |  |  |  |  |  |  |  |  |  |  |  |
| Nonagenarians | 28 | 4 |  |  |  |  | 12 | 4 |  |  |  |  | 24 | 2 |  |  |  |  | 11 | 2 |  |  |  |  |
| Control | 88 | 10 | 0.972 | 0.795 | 0.231 | 2.735 | 40 | 9 | 0.829 | 0.675 | 0.176 | 2.585 | 51 | 11 | 0.377 | 2.588 | 0.532 | 12.601 | 20 | 11 | 0.331 | 3.025 | 0.566 | 16.177 |
| Lipids（+）+FBG（-）+BMI（-） |  |  |  |  |  |  |  |  |  |  |  |  |  |  |  |  |  |  |  |  |  |  |  |  |
| Nonagenarians | 150 | 28 |  |  |  |  | 63 | 26 |  |  |  |  | 50 | 2 |  |  |  |  | 24 | 2 |  |  |  |  |
| Control | 513 | 69 | 0.175 | 0.721 | 0.448 | 1.159 | 227 | 64 | 0.161 | 0.683 | 0.4 | 1.166 | 216 | 24 | 0.253 | 2.778 | 0.636 | 12.141 | 97 | 23 | 0.262 | 2.845 | 0.627 | 12.911 |
| Lipids（-）+FBG（+）+BMI（+） |  |  |  |  |  |  |  |  |  |  |  |  |  |  |  |  |  |  |  |  |  |  |  |  |
| Nonagenarians | 10 | 2 |  |  |  |  | 4 | 2 |  |  |  |  | 6 | 0 |  |  |  |  | 3 | 0 |  |  |  |  |
| Control | 34 | 6 | 1.000 | 0.882 | 0.154 | 5.071 | 15 | 5 | 1.000 | 0.667 | 0.092 | 4.81 | 22 | 2 | 1.000 | 0.913 | 0.082 | 10.228 | 10 | 2 | 1.000 | 1.091 | .086 | 13.778 |
| Lipids（+）+FBG（-）+BMI（+） |  |  |  |  |  |  |  |  |  |  |  |  |  |  |  |  |  |  |  |  |  |  |  |  |
| Nonagenarians | 125 | 15 |  |  |  |  | 56 | 14 |  |  |  |  | 28 | 10 |  |  |  |  | 10 | 9 |  |  |  |  |
| Control | 247 | 47 | 0.142 | 1.586 | 0.853 | 2.947 | 103 | 44 | 0.122 | 1.709 | 0.862 | 3.386 | 116 | 16 | 0.032 | 0.386 | 0.158 | 0.942 | 53 | 13 | 0.033 | 0.273 | .092 | 0.807 |
| Lipids（+）+FBG（+）+BMI（-） |  |  |  |  |  |  |  |  |  |  |  |  |  |  |  |  |  |  |  |  |  |  |  |  |
| Nonagenarians | 9 | 1 |  |  |  |  | 4 | 1 |  |  |  |  | 14 | 0 |  |  |  |  | 7 | 0 |  |  |  |  |
| Control | 55 | 7 | 1.000 | 1.145 | 0.126 | 10.448 | 24 | 7 | 1.000 | 1.167 | 0.112 | 12.202 | 25 | 7 | 0.276 | 4.615 | 0.525 | 40.577 | 10 | 6 | 0.297 | 5.091 | .518 | 50.004 |
| Lipids（+）+FBG（+）+BMI（+） |  |  |  |  |  |  |  |  |  |  |  |  |  |  |  |  |  |  |  |  |  |  |  |  |
| Nonagenarians | 14 | 2 |  |  |  |  | 6 | 2 |  |  |  |  | 10 | 2 |  |  |  |  | 4 | 2 |  |  |  |  |
| Control | 79 | 7 | 0.933 | 0.62 | 0.117 | 3.298 | 36 | 7 | 0.929 | 0.583 | 0.097 | 3.506 | 16 | 4 | 1.000 | 1.25 | 0.192 | 8.129 | 6 | 4 | 1.000 | 1.333 | 0.161 | 11.075 |
| Longevity/Control |  |  |  |  |  |  |  |  |  |  |  |  |  |  |  |  |  |  |  |  |  |  |  |  |
| Lipids（-）+FBG（-）+BMI（-） |  |  |  |  |  |  |  |  |  |  |  |  |  |  |  |  |  |  |  |  |  |  |  |  |
| Longevity | 803 | 117 |  |  |  |  | 349 | 111 |  |  |  |  | 535 | 59 |  |  |  |  | 244 | 53 |  |  |  |  |
| Control | 1197 | 159 | 0.477 | 0.912 | 0.707 | 1.176 | 528 | 150 | 0.429 | 0.893 | 0.675 | 1.182 | 583 | 81 | 0.202 | 1.26 | 0.883 | 1.797 | 256 | 76 | 0.118 | 1.367 | .923 | 2.023 |
| Lipids（-）+FBG（-）+BMI（+） |  |  |  |  |  |  |  |  |  |  |  |  |  |  |  |  |  |  |  |  |  |  |  |  |
| Longevity | 343 | 47 |  |  |  |  | 154 | 41 |  |  |  |  | 273 | 37 |  |  |  |  | 122 | 33 |  |  |  |  |
| Control | 353 | 57 | 0.436 | 1.178 | 0.779 | 1.782 | 156 | 49 | 0.491 | 1.18 | 0.737 | 1.889 | 241 | 39 | 0.471 | 1.194 | 0.737 | 1.933 | 102 | 38 | 0.240 | 1.377 | 0.806 | 2.353 |
| Lipids（-）+FBG（+）+BMI（-） |  |  |  |  |  |  |  |  |  |  |  |  |  |  |  |  |  |  |  |  |  |  |  |  |
| Longevity | 69 | 7 |  |  |  |  | 31 | 7 |  |  |  |  | 46 | 6 |  |  |  |  | 21 | 5 |  |  |  |  |
| Control | 88 | 10 | 0.827 | 1.12 | 0.406 | 3.094 | 40 | 9 | 0.995 | 0.996 | 0.334 | 2.974 | 51 | 11 | 0.354 | 1.654 | 0.566 | 4.829 | 20 | 11 | 0.174 | 2.31 | 0.681 | 7.838 |
| Lipids（+）+FBG（-）+BMI（-） |  |  |  |  |  |  |  |  |  |  |  |  |  |  |  |  |  |  |  |  |  |  |  |  |
| Longevity | 361 | 45 |  |  |  |  | 161 | 42 |  |  |  |  | 202 | 10 |  |  |  |  | 96 | 10 |  |  |  |  |
| Control | 513 | 69 | 0.709 | 1.079 | 0.724 | 1.608 | 227 | 64 | 0.728 | 1.081 | 0.697 | 1.676 | 216 | 24 | 0.034 | 2.244 | 1.047 | 4.81 | 97 | 23 | 0.039 | 2.276 | 1.029 | 5.037 |
| Lipids（-）+FBG（+）+BMI（+） |  |  |  |  |  |  |  |  |  |  |  |  |  |  |  |  |  |  |  |  |  |  |  |  |
| Longevity | 33 | 3 |  |  |  |  | 15 | 3 |  |  |  |  | 28 | 0 |  |  |  |  | 14 | 0 |  |  |  |  |
| Control | 34 | 6 | 0.587 | 1.941 | 0.448 | 8.412 | 15 | 5 | 0.818 | 1.667 | 0.336 | 8.258 | 22 | 2 | 0.504 | 3.783 | 0.369 | 38.815 | 10 | 2 | 0.495 | 4.091 | .374 | 44.788 |
| Lipids（+）+FBG（-）+BMI（+） |  |  |  |  |  |  |  |  |  |  |  |  |  |  |  |  |  |  |  |  |  |  |  |  |
| Longevity | 228 | 36 |  |  |  |  | 98 | 34 |  |  |  |  | 114 | 18 |  |  |  |  | 50 | 16 |  |  |  |  |
| Control | 247 | 47 | 0.436 | 1.205 | 0.753 | 1.928 | 103 | 44 | 0.438 | 1.231 | 0.728 | 2.084 | 116 | 16 | 0.713 | 0.874 | 0.425 | 1.797 | 53 | 13 | 0.528 | 0.767 | .335 | 1.754 |
| Lipids（+）+FBG（+）+BMI（-） |  |  |  |  |  |  |  |  |  |  |  |  |  |  |  |  |  |  |  |  |  |  |  |  |
| Longevity | 28 | 2 |  |  |  |  | 13 | 2 |  |  |  |  | 30 | 0 |  |  |  |  | 15 | 0 |  |  |  |  |
| Control | 55 | 7 | 0.745 | 1.782 | 0.347 | 9.149 | 24 | 7 | 0.730 | 1.896 | 0.343 | 10.485 | 25 | 7 | 0.040 | 9.538 | 1.119 | 81.331 | 10 | 6 | 0.055 | 10.182 | 1.093 | 94.831 |
| Lipids（+）+FBG（+）+BMI（+） |  |  |  |  |  |  |  |  |  |  |  |  |  |  |  |  |  |  |  |  |  |  |  |  |
| Longevity | 30 | 2 |  |  |  |  | 14 | 2 |  |  |  |  | 17 | 3 |  |  |  |  | 7 | 3 |  |  |  |  |
| Control | 79 | 7 | 1.000 | 1.329 | 0.261 | 6.761 | 36 | 7 | 1.000 | 1.361 | 0.252 | 7.365 | 16 | 4 | 1.000 | 1.417 | 0.273 | 7.342 | 6 | 4 | 1.000 | 1.556 | 0.244 | 9.913 |
