# Supplementary material for: Identification of cardiovascular health gene variants related to longevity in a Chinese population
Source: Aging (Albany NY). 2020 Sep 7;12(17):16775–802. doi: 10.18632/aging.103396 (PMC7521493; doi:10.18632/aging.103396)
Supplement: Supplementary Table 6 [file aging-12-103396-s005..docx]

**Supplementary Table 6. Stratification analysis of metabolic phenotype with polymorphism of TFPI rs7586970 and ADAMTS7 rs3825807.**

|  | rs7586970*rs3825807 |  |  |  |  |  |
| --- | --- | --- | --- | --- | --- | --- |
| Centenarians/Nonagenarians | TTAA | CCGG+TTGG+TTAG+TCAA+TCAG+CCAG+CCAA+TCGG | p | OR | 95%CI |  |
| Lipids（-）+FBG（-）+BMI（-） |  |  |  |  |  |  |
| Centenarians | 89 | 27 |  |  |  |  |
| Nonagenarians | 60 | 39 | 0.011 | 2.143 | 1.188 | 3.865 |
| Lipids（-）+FBG（-）+BMI（+） |  |  |  |  |  |  |
| Centenarians | 38 | 16 |  |  |  |  |
| Nonagenarians | 20 | 27 | 0.005 | 3.206 | 1.409 | 7.294 |
| Lipids（-）+FBG（+）+BMI（-） |  |  |  |  |  |  |
| Centenarians | 9 | 2 |  |  |  |  |
| Nonagenarians | 4 | 0 | 1.000 | 0.667 | 0.054 | 8.161 |
| Lipids（+）+FBG（-）+BMI（-） |  |  |  |  |  |  |
| Centenarians | 39 | 17 |  |  |  |  |
| Nonagenarians | 15 | 8 | 0.701 | 1.224 | 0.437 | 3.427 |
| Lipids（-）+FBG（+）+BMI（+） |  |  |  |  |  |  |
| Centenarians | 5 | 0 |  |  |  |  |
| Nonagenarians | 2 | 2 | 0.431 | 6 | 0.422 | 85.248 |
| Lipids（+）+FBG（-）+BMI（+） |  |  |  |  |  |  |
| Centenarians | 16 | 11 |  |  |  |  |
| Nonagenarians | 17 | 7 | 0.388 | 0.599 | 0.186 | 1.926 |
| Lipids（+）+FBG（+）+BMI（-） |  |  |  |  |  |  |
| Centenarians | 5 | 0 |  |  |  |  |
| Nonagenarians | 1 | 0 | 1.000 | 3 | 0.122 | 73.642 |
| Lipids（+）+FBG（+）+BMI（+） |  |  |  |  |  |  |
| Centenarians | 4 | 0 |  |  |  |  |
| Nonagenarians | 0 | 4 | 0.083 | 25 | 1.2 | 520.734 |
| Centenarians/Control |  |  |  |  |  |  |
| Lipids（-）+FBG（-）+BMI（-） |  |  |  |  |  |  |
| Centenarians | 89 | 27 |  |  |  |  |
| Control | 143 | 88 | 0.006 | 2.028 | 1.223 | 3.364 |
| Lipids（-）+FBG（-）+BMI（+） |  |  |  |  |  |  |
| Centenarians | 38 | 16 |  |  |  |  |
| Control | 48 | 22 | 0.829 | 1.089 | 0.503 | 2.355 |
| Lipids（-）+FBG（+）+BMI（-） |  |  |  |  |  |  |
| Centenarians | 9 | 2 |  |  |  |  |
| Control | 8 | 11 | 0.083 | 6.188 | 1.041 | 36.779 |
| Lipids（+）+FBG（-）+BMI（-） |  |  |  |  |  |  |
| Centenarians | 39 | 17 |  |  |  |  |
| Control | 59 | 39 | 0.241 | 1.516 | 0.754 | 3.05 |
| Lipids（-）+FBG（+）+BMI（+） |  |  |  |  |  |  |
| Centenarians | 5 | 0 |  |  |  |  |
| Control | 4 | 4 | 0.317 | 6 | 0.516 | 69.754 |
| Lipids（+）+FBG（-）+BMI（+） |  |  |  |  |  |  |
| Centenarians | 16 | 11 |  |  |  |  |
| Control | 31 | 22 | 0.947 | 1.032 | 0.402 | 2.649 |
| Lipids（+）+FBG（+）+BMI（-） |  |  |  |  |  |  |
| Centenarians | 5 | 0 |  |  |  |  |
| Control | 7 | 7 | 0.250 | 6 | 0.582 | 61.842 |
| Lipids（+）+FBG（+）+BMI（+） |  |  |  |  |  |  |
| Centenarians | 4 | 0 |  |  |  |  |
| Control | 14 | 5 | 0.953 | 2 | 0.191 | 20.898 |
| Nonagenarians/Control |  |  |  |  |  |  |
| Lipids（-）+FBG（-）+BMI（-） |  |  |  |  |  |  |
| Nonagenarians | 60 | 39 |  |  |  |  |
| Control | 143 | 88 | 0.824 | 0.947 | 0.584 | 1.534 |
| Lipids（-）+FBG（-）+BMI（+） |  |  |  |  |  |  |
| Nonagenarians | 20 | 27 |  |  |  |  |
| Control | 48 | 22 | 0.005 | 0.34 | 0.158 | 0.731 |
| Lipids（-）+FBG（+）+BMI（-） |  |  |  |  |  |  |
| Nonagenarians | 4 | 0 |  |  |  |  |
| Control | 8 | 11 | 0.198 | 6.667 | 0.659 | 67.463 |
| Lipids（+）+FBG（-）+BMI（-） |  |  |  |  |  |  |
| Nonagenarians | 15 | 8 |  |  |  |  |
| Control | 59 | 39 | 0.657 | 1.239 | 0.48 | 3.2 |
| Lipids（-）+FBG（+）+BMI（+） |  |  |  |  |  |  |
| Nonagenarians | 2 | 2 |  |  |  |  |
| Control | 4 | 4 | 1.000 | 1 | 0.091 | 11.028 |
| Lipids（+）+FBG（-）+BMI（+） |  |  |  |  |  |  |
| Nonagenarians | 17 | 7 |  |  |  |  |
| Control | 31 | 22 | 0.301 | 1.724 | 0.612 | 4.857 |
| Lipids（+）+FBG（+）+BMI（-） |  |  |  |  |  |  |
| Nonagenarians | 1 | 0 |  |  |  |  |
| Control | 7 | 7 | 1.000 | 2 | 0.15 | 26.734 |
| Lipids（+）+FBG（+）+BMI（+） |  |  |  |  |  |  |
| Nonagenarians | 0 | 4 |  |  |  |  |
| Control | 14 | 5 | 0.053 | 0.08 | 0.008 | 0.836 |
| Longevity/Control |  |  |  |  |  |  |
| Lipids（-）+FBG（-）+BMI（-） |  |  |  |  |  |  |
| Longevity | 149 | 66 |  |  |  |  |
| Control | 143 | 88 | 0.101 | 1.389 | 0.938 | 2.058 |
| Lipids（-）+FBG（-）+BMI（+） |  |  |  |  |  |  |
| Longevity | 58 | 43 |  |  |  |  |
| Control | 48 | 22 | 0.140 | 0.618 | 0.326 | 1.173 |
| Lipids（-）+FBG（+）+BMI（-） |  |  |  |  |  |  |
| Longevity | 13 | 2 |  |  |  |  |
| Control | 8 | 11 | 0.008 | 8.938 | 1.561 | 51.184 |
| Lipids（+）+FBG（-）+BMI（-） |  |  |  |  |  |  |
| Longevity | 54 | 25 |  |  |  |  |
| Control | 59 | 39 | 0.262 | 1.428 | 0.766 | 2.663 |
| Lipids（-）+FBG（+）+BMI（+） |  |  |  |  |  |  |
| Longevity | 7 | 2 |  |  |  |  |
| Control | 4 | 4 | 0.492 | 3.5 | 0.431 | 28.447 |
| Lipids（+）+FBG（-）+BMI（+） |  |  |  |  |  |  |
| Longevity | 33 | 18 |  |  |  |  |
| Control | 31 | 22 | 0.515 | 1.301 | 0.589 | 2.874 |
| Lipids（+）+FBG（+）+BMI（-） |  |  |  |  |  |  |
| Longevity | 6 | 0 |  |  |  |  |
| Control | 7 | 7 | 0.180 | 7 | 0.693 | 70.743 |
| Lipids（+）+FBG（+）+BMI（+） |  |  |  |  |  |  |
| Longevity | 4 | 4 |  |  |  |  |
| Control | 14 | 5 | 0.456 | 0.357 | 0.064 | 1.997 |
