# Supplementary material for: Identification of cardiovascular health gene variants related to longevity in a Chinese population
Source: Aging (Albany NY). 2020 Sep 7;12(17):16775–802. doi: 10.18632/aging.103396 (PMC7521493; doi:10.18632/aging.103396)
Supplement: Supplementary Table 7 [file aging-12-103396-s003..docx]

**Supplementary Table 7. Stratification analysis of metabolic phenotype with polymorphism of TFPI rs7586970, ADAMTS7 rs3825807 and APOEε3.**

| Centenarians/Nonagenarians | rs7586970*APOEε3 |  |  |  |  |  | rs3825807*APOEε3 |  |  |  |  |  |
| --- | --- | --- | --- | --- | --- | --- | --- | --- | --- | --- | --- | --- |
| Lipids（-）+FBG（-）+BMI（-） | TTε3ε3 | CCε3ε3 + TCε3ε3 | p | OR | 95%CI |  | AAε3ε3 | AGε3ε3+GGε3ε3 | p | OR | 95%CI |  |
| Centenarians | 105 | 27 |  |  |  |  | 87 | 18 |  |  |  |  |
| Nonagenarians | 117 | 52 | 0.044 | 1.728 | 1.013 | 2.949 | 62 | 20 | 0.222 | 1.559 | 0.763 | 3.188 |
| Lipids（-）+FBG（-）+BMI（+） |  |  |  |  |  |  |  |  |  |  |  |  |
| Centenarians | 65 | 12 |  |  |  |  | 46 | 11 |  |  |  |  |
| Nonagenarians | 46 | 24 | 0.008 | 2.826 | 1.284 | 6.222 | 34 | 16 | 0.131 | 1.968 | 0.811 | 4.775 |
| Lipids（-）+FBG（+）+BMI（-） |  |  |  |  |  |  |  |  |  |  |  |  |
| Centenarians | 10 | 2 |  |  |  |  | 4 | 2 |  |  |  |  |
| Nonagenarians | 8 | 2 | 1.000 | 1.25 | 0.143 | 10.94 | 4 | 0 | 0.798 | 0.333 | 0.025 | 4.401 |
| Lipids（+）+FBG（-）+BMI（-） |  |  |  |  |  |  |  |  |  |  |  |  |
| Centenarians | 59 | 8 |  |  |  |  | 36 | 4 |  |  |  |  |
| Nonagenarians | 42 | 19 | 0.008 | 3.336 | 1.335 | 8.338 | 14 | 2 | 1.000 | 1.286 | 0.211 | 7.826 |
| Lipids（-）+FBG（+）+BMI（+） |  |  |  |  |  |  |  |  |  |  |  |  |
| Centenarians | 8 | 0 |  |  |  |  | 5 | 0 |  |  |  |  |
| Nonagenarians | 3 | 2 | 0.322 | 6.75 | 0.526 | 86.561 | 1 | 0 | 1.000 | 3 | 0.122 | 73.642 |
| Lipids（+）+FBG（-）+BMI（+） |  |  |  |  |  |  |  |  |  |  |  |  |
| Centenarians | 27 | 12 |  |  |  |  | 28 | 4 |  |  |  |  |
| Nonagenarians | 34 | 8 | 0.222 | 0.529 | 0.189 | 1.479 | 8 | 7 | 0.027 | 6.125 | 1.425 | 26.328 |
| Lipids（+）+FBG（+）+BMI（-） |  |  |  |  |  |  |  |  |  |  |  |  |
| Centenarians | 9 | 0 |  |  |  |  | 4 | 0 |  |  |  |  |
| Nonagenarians | 2 | 1 | 0.214 | 6.667 | 0.437 | 101.732 | 3 | 0 | 1.000 | 1.25 | 0.058 | 26.869 |
| Lipids（+）+FBG（+）+BMI（+） |  |  |  |  |  |  |  |  |  |  |  |  |
| Centenarians | 2 | 0 |  |  |  |  | 2 | 1 |  |  |  |  |
| Nonagenarians | 5 | 1 | 1.000 | 1 | 0.063 | 15.988 | 3 | 2 | 1.000 | 1.333 | 0.067 | 26.618 |
| Centenarians/Control |  |  |  |  |  |  |  |  |  |  |  |  |
| Lipids（-）+FBG（-）+BMI（-） |  |  |  |  |  |  |  |  |  |  |  |  |
| Centenarians | 105 | 27 |  |  |  |  | 87 | 18 |  |  |  |  |
| Control | 360 | 104 | 0.631 | 1.123 | 0.698 | 1.808 | 171 | 52 | 0.259 | 1.47 | 0.811 | 2.665 |
| Lipids（-）+FBG（-）+BMI（+） |  |  |  |  |  |  |  |  |  |  |  |  |
| Centenarians | 65 | 12 |  |  |  |  | 46 | 11 |  |  |  |  |
| Control | 93 | 37 | 0.035 | 2.155 | 1.045 | 4.446 | 70 | 28 | 0.200 | 1.673 | 0.759 | 3.687 |
| Lipids（-）+FBG（+）+BMI（-） |  |  |  |  |  |  |  |  |  |  |  |  |
| Centenarians | 10 | 2 |  |  |  |  | 4 | 2 |  |  |  |  |
| Control | 27 | 5 | 1.000 | 0.926 | 0.154 | 5.563 | 14 | 8 | 1.000 | 1.143 | 0.17 | 7.693 |
| Lipids（+）+FBG（-）+BMI（-） |  |  |  |  |  |  |  |  |  |  |  |  |
| Centenarians | 59 | 8 |  |  |  |  | 36 | 4 |  |  |  |  |
| Control | 151 | 39 | 0.118 | 1.905 | 0.841 | 4.316 | 64 | 18 | 0.107 | 2.531 | 0.795 | 8.057 |
| Lipids（-）+FBG（+）+BMI（+） |  |  |  |  |  |  |  |  |  |  |  |  |
| Centenarians | 8 | 0 |  |  |  |  | 5 | 0 |  |  |  |  |
| Control | 10 | 3 | 0.610 | 3.273 | 0.308 | 34.722 | 3 | 0 | 1.000 | 1.5 | 0.071 | 31.575 |
| Lipids（+）+FBG（-）+BMI（+） |  |  |  |  |  |  |  |  |  |  |  |  |
| Centenarians | 27 | 12 |  |  |  |  | 28 | 4 |  |  |  |  |
| Control | 63 | 27 | 0.930 | 0.964 | 0.426 | 2.18 | 44 | 9 | 0.806 | 1.432 | 0.402 | 5.096 |
| Lipids（+）+FBG（+）+BMI（-） |  |  |  |  |  |  |  |  |  |  |  |  |
| Centenarians | 9 | 0 |  |  |  |  | 4 | 0 |  |  |  |  |
| Control | 13 | 4 | 0.507 | 3.571 | 0.36 | 35.454 | 7 | 5 | 0.539 | 3.75 | 0.342 | 41.081 |
| Lipids（+）+FBG（+）+BMI（+） |  |  |  |  |  |  |  |  |  |  |  |  |
| Centenarians | 2 | 0 |  |  |  |  | 2 | 1 |  |  |  |  |
| Control | 23 | 5 | 1.000 | 0.75 | 0.066 | 8.55 | 3 | 2 | 1.000 | 1.333 | 0.067 | 26.618 |
| Nonagenarians/Control |  |  |  |  |  |  |  |  |  |  |  |  |
| Lipids（-）+FBG（-）+BMI（-） |  |  |  |  |  |  |  |  |  |  |  |  |
| Nonagenarians | 117 | 52 |  |  |  |  | 62 | 20 |  |  |  |  |
| Control | 360 | 104 | 0.031 | 0.65 | 0.439 | 0.963 | 171 | 52 | 0.845 | 0.943 | 0.522 | 1.704 |
| Lipids（-）+FBG（-）+BMI（+） |  |  |  |  |  |  |  |  |  |  |  |  |
| Nonagenarians | 46 | 24 |  |  |  |  | 34 | 16 |  |  |  |  |
| Control | 93 | 37 | 0.393 | 0.763 | 0.409 | 1.422 | 70 | 28 | 0.666 | 0.85 | 0.406 | 1.779 |
| Lipids（-）+FBG（+）+BMI（-） |  |  |  |  |  |  |  |  |  |  |  |  |
| Nonagenarians | 8 | 2 |  |  |  |  | 4 | 0 |  |  |  |  |
| Control | 27 | 5 | 1.000 | 0.741 | 0.12 | 4.571 | 14 | 8 | 0.628 | 3 | 0.301 | 29.94 |
| Lipids（+）+FBG（-）+BMI（-） |  |  |  |  |  |  |  |  |  |  |  |  |
| Nonagenarians | 42 | 19 |  |  |  |  | 14 | 2 |  |  |  |  |
| Control | 151 | 39 | 0.087 | 0.571 | 0.299 | 1.09 | 64 | 18 | 0.604 | 1.969 | 0.409 | 9.474 |
| Lipids（-）+FBG（+）+BMI（+） |  |  |  |  |  |  |  |  |  |  |  |  |
| Nonagenarians | 3 | 2 |  |  |  |  | 1 | 0 |  |  |  |  |
| Control | 10 | 3 | 0.896 | 0.45 | 0.05 | 4.085 | 3 | 0 | 1.000 | 0.5 | 0.019 | 12.898 |
| Lipids（+）+FBG（-）+BMI（+） |  |  |  |  |  |  |  |  |  |  |  |  |
| Nonagenarians | 34 | 8 |  |  |  |  | 8 | 7 |  |  |  |  |
| Control | 63 | 27 | 0.184 | 1.821 | 0.746 | 4.446 | 44 | 9 | 0.041 | 0.234 | 0.067 | 0.81 |
| Lipids（+）+FBG（+）+BMI（-） |  |  |  |  |  |  |  |  |  |  |  |  |
| Nonagenarians | 2 | 1 |  |  |  |  | 3 | 0 |  |  |  |  |
| Control | 13 | 4 | 1.000 | 0.615 | 0.044 | 8.703 | 7 | 5 | 0.712 | 3 | 0.263 | 34.198 |
| Lipids（+）+FBG（+）+BMI（+） |  |  |  |  |  |  |  |  |  |  |  |  |
| Nonagenarians | 5 | 1 |  |  |  |  | 3 | 2 |  |  |  |  |
| Control | 23 | 5 | 1.000 | 1.087 | 0.103 | 11.452 | 3 | 2 | 1.000 | 1 | 0.08 | 12.557 |
| Longevity/Control |  |  |  |  |  |  |  |  |  |  |  |  |
| Lipids（-）+FBG（-）+BMI（-） |  |  |  |  |  |  |  |  |  |  |  |  |
| Longevity | 222 | 79 |  |  |  |  | 140 | 38 |  |  |  |  |
| Control | 360 | 104 | 0.225 | 0.812 | 0.58 | 1.137 | 171 | 52 | 0.638 | 1.12 | 0.697 | 1.8 |
| Lipids（-）+FBG（-）+BMI（+） |  |  |  |  |  |  |  |  |  |  |  |  |
| Longevity | 113 | 36 |  |  |  |  | 80 | 27 |  |  |  |  |
| Control | 93 | 37 | 0.415 | 1.249 | 0.732 | 2.131 | 70 | 28 | 0.590 | 1.185 | 0.639 | 2.2 |
| Lipids（-）+FBG（+）+BMI（-） |  |  |  |  |  |  |  |  |  |  |  |  |
| Longevity | 18 | 4 |  |  |  |  | 8 | 2 |  |  |  |  |
| Control | 27 | 5 | 1.000 | 0.833 | 0.197 | 3.53 | 14 | 8 | 0.607 | 2.286 | 0.387 | 13.505 |
| Lipids（+）+FBG（-）+BMI（-） |  |  |  |  |  |  |  |  |  |  |  |  |
| Longevity | 101 | 27 |  |  |  |  | 50 | 6 |  |  |  |  |
| Control | 151 | 39 | 0.903 | 0.966 | 0.557 | 1.677 | 64 | 18 | 0.087 | 2.344 | 0.866 | 6.341 |
| Lipids（-）+FBG（+）+BMI（+） |  |  |  |  |  |  |  |  |  |  |  |  |
| Longevity | 11 | 2 |  |  |  |  | 6 | 0 |  |  |  |  |
| Control | 10 | 3 | 1.000 | 1.65 | 0.227 | 11.993 | 3 | 0 | 1.000 | 1.75 | 0.084 | 36.287 |
| Lipids（+）+FBG（-）+BMI（+） |  |  |  |  |  |  |  |  |  |  |  |  |
| Longevity | 61 | 20 |  |  |  |  | 36 | 11 |  |  |  |  |
| Control | 63 | 27 | 0.438 | 1.307 | 0.664 | 2.572 | 44 | 9 | 0.423 | 0.669 | 0.25 | 1.792 |
| Lipids（+）+FBG（+）+BMI（-） |  |  |  |  |  |  |  |  |  |  |  |  |
| Longevity | 11 | 1 |  |  |  |  | 7 | 0 |  |  |  |  |
| Control | 13 | 4 | 0.570 | 3.385 | 0.328 | 34.919 | 7 | 5 | 0.250 | 6 | 0.582 | 61.842 |
| Lipids（+）+FBG（+）+BMI（+） |  |  |  |  |  |  |  |  |  |  |  |  |
| Longevity | 7 | 1 |  |  |  |  | 5 | 3 |  |  |  |  |
| Control | 23 | 5 | 1.000 | 1.522 | 0.151 | 15.296 | 3 | 2 | 1.000 | 1.111 | 0.112 | 10.986 |
