# Supplementary material for: Identification of cardiovascular health gene variants related to longevity in a Chinese population
Source: Aging (Albany NY). 2020 Sep 7;12(17):16775–802. doi: 10.18632/aging.103396 (PMC7521493; doi:10.18632/aging.103396)
Supplement: Supplementary Table 8 [file aging-12-103396-s004..docx]

**Supplementary Table 8. Stratification analysis of metabolic phenotype with polymorphism of TFPI rs7586970 and ADAMTS7 rs3825807 and APOEε3.**

| Centenarians/Nonagenarians | rs7586970*rs3825807*APOEε3 |  |  |  |  |  |
| --- | --- | --- | --- | --- | --- | --- |
| Lipids（-）+FBG（-）+BMI（-） | TTAAε3ε3 | TTAGε3ε3+TTGGε3ε3+TCAAε3ε3+TCAGε3ε3+TCGGε3ε3+CCAAε3ε3+CCAGε3ε3+CCGGε3ε3 | p | OR | 95%CI |  |
| Centenarians | 48 | 0 |  |  |  |  |
| Nonagenarians | 40 | 0 | 0.030 | 2.25 | 1.076 | 4.705 |
| Lipids（-）+FBG（-）+BMI（+） |  |  |  |  |  |  |
| Centenarians | 26 | 0 |  |  |  |  |
| Nonagenarians | 15 | 0 | 0.026 | 2.773 | 1.121 | 6.859 |
| Lipids（-）+FBG（+）+BMI（-） |  |  |  |  |  |  |
| Centenarians | 5 | 6 |  |  |  |  |
| Nonagenarians | 1 | 3 | 0.905 | 2.5 | 0.194 | 32.194 |
| Lipids（+）+FBG（-）+BMI（-） |  |  |  |  |  |  |
| Centenarians | 20 | 33 |  |  |  |  |
| Nonagenarians | 7 | 10 | 0.800 | 0.866 | 0.284 | 2.638 |
| Lipids（-）+FBG（+）+BMI（+） |  |  |  |  |  |  |
| Centenarians | 3 | 0 |  |  |  |  |
| Nonagenarians | 1 | 0 | 0.519 | 6 | 0.354 | 101.568 |
| Lipids（+）+FBG（-）+BMI（+） |  |  |  |  |  |  |
| Centenarians | 4 | 23 |  |  |  |  |
| Nonagenarians | 9 | 15 | 0.064 | 0.29 | 0.075 | 1.113 |
| Lipids（+）+FBG（+）+BMI（-） |  |  |  |  |  |  |
| Centenarians | 5 | 0 |  |  |  |  |
| Nonagenarians | 0 | 1 | 0.183 | 12 | 0.489 | 294.569 |
| Lipids（+）+FBG（+）+BMI（+） |  |  |  |  |  |  |
| Centenarians | 0 | 0 |  |  |  |  |
| Nonagenarians | 0 | 0 | 1.000 | 4 | 0.117 | 136.957 |
| Centenarians/Control |  |  |  |  |  |  |
| Lipids（-）+FBG（-）+BMI（-） |  |  |  |  |  |  |
| Centenarians | 48 | 0 |  |  |  |  |
| Control | 94 | 0 | 0.043 | 1.947 | 1.015 | 3.733 |
| Lipids（-）+FBG（-）+BMI（+） |  |  |  |  |  |  |
| Centenarians | 26 | 0 |  |  |  |  |
| Control | 28 | 0 | 0.909 | 1.052 | 0.438 | 2.527 |
| Lipids（-）+FBG（+）+BMI（-） |  |  |  |  |  |  |
| Centenarians | 5 | 6 |  |  |  |  |
| Control | 4 | 15 | 0.321 | 3.125 | 0.618 | 15.794 |
| Lipids（+）+FBG（-）+BMI（-） |  |  |  |  |  |  |
| Centenarians | 20 | 33 |  |  |  |  |
| Control | 44 | 47 | 0.216 | 0.647 | 0.324 | 1.292 |
| Lipids（-）+FBG（+）+BMI（+） |  |  |  |  |  |  |
| Centenarians | 3 | 0 |  |  |  |  |
| Control | 3 | 0 | 0.620 | 4 | 0.299 | 53.468 |
| Lipids（+）+FBG（-）+BMI（+） |  |  |  |  |  |  |
| Centenarians | 4 | 23 |  |  |  |  |
| Control | 18 | 35 | 0.070 | 0.338 | 0.101 | 1.128 |
| Lipids（+）+FBG（+）+BMI（-） |  |  |  |  |  |  |
| Centenarians | 5 | 0 |  |  |  |  |
| Control | 4 | 10 | 0.051 | 13.2 | 1.239 | 140.679 |
| Lipids（+）+FBG（+）+BMI（+） |  |  |  |  |  |  |
| Centenarians | 0 | 0 |  |  |  |  |
| Control | 8 | 0 | 1.000 | 0.556 | 0.028 | 10.933 |
| Nonagenarians/Control |  |  |  |  |  |  |
| Lipids（-）+FBG（-）+BMI（-） |  |  |  |  |  |  |
| Nonagenarians | 40 | 0 |  |  |  |  |
| Control | 94 | 0 | 0.620 | 0.865 | 0.488 | 1.534 |
| Lipids（-）+FBG（-）+BMI（+） |  |  |  |  |  |  |
| Nonagenarians | 15 | 0 |  |  |  |  |
| Control | 28 | 0 | 0.030 | 0.379 | 0.157 | 0.917 |
| Lipids（-）+FBG（+）+BMI（-） |  |  |  |  |  |  |
| Nonagenarians | 1 | 3 |  |  |  |  |
| Control | 4 | 15 | 1.000 | 1.25 | 0.101 | 15.499 |
| Lipids（+）+FBG（-）+BMI（-） |  |  |  |  |  |  |
| Nonagenarians | 7 | 10 |  |  |  |  |
| Control | 44 | 47 | 0.586 | 0.748 | 0.262 | 2.136 |
| Lipids（-）+FBG（+）+BMI（+） |  |  |  |  |  |  |
| Nonagenarians | 1 | 0 |  |  |  |  |
| Control | 3 | 0 | 1.000 | 0.5 | 0.028 | 8.952 |
| Lipids（+）+FBG（-）+BMI（+） |  |  |  |  |  |  |
| Nonagenarians | 9 | 15 |  |  |  |  |
| Control | 18 | 35 | 0.763 | 1.167 | 0.428 | 3.181 |
| Lipids（+）+FBG（+）+BMI（-） |  |  |  |  |  |  |
| Nonagenarians | 0 | 1 |  |  |  |  |
| Control | 4 | 10 | 1.000 | 1.1 | 0.08 | 15.153 |
| Lipids（+）+FBG（+）+BMI（+） |  |  |  |  |  |  |
| Nonagenarians | 0 | 0 |  |  |  |  |
| Control | 8 | 0 | 0.238 | 0.139 | 0.012 | 1.608 |
| Longevity/Control |  |  |  |  |  |  |
| Lipids（-）+FBG（-）+BMI（-） |  |  |  |  |  |  |
| Longevity | 88 | 0 |  |  |  |  |
| Control | 94 | 0 | 0.378 | 1.241 | 0.768 | 2.008 |
| Lipids（-）+FBG（-）+BMI（+） |  |  |  |  |  |  |
| Longevity | 41 | 0 |  |  |  |  |
| Control | 28 | 0 | 0.236 | 0.638 | 0.303 | 1.345 |
| Lipids（-）+FBG（+）+BMI（-） |  |  |  |  |  |  |
| Longevity | 6 | 9 |  |  |  |  |
| Control | 4 | 15 | 0.409 | 2.5 | 0.552 | 11.33 |
| Lipids（+）+FBG（-）+BMI（-） |  |  |  |  |  |  |
| Longevity | 27 | 43 |  |  |  |  |
| Control | 44 | 47 | 0.215 | 0.671 | 0.356 | 1.263 |
| Lipids（-）+FBG（+）+BMI（+） |  |  |  |  |  |  |
| Longevity | 4 | 0 |  |  |  |  |
| Control | 3 | 0 | 1.000 | 2 | 0.194 | 20.614 |
| Lipids（+）+FBG（-）+BMI（+） |  |  |  |  |  |  |
| Longevity | 13 | 38 |  |  |  |  |
| Control | 18 | 35 | 0.345 | 0.665 | 0.285 | 1.554 |
| Lipids（+）+FBG（+）+BMI（-） |  |  |  |  |  |  |
| Longevity | 5 | 1 |  |  |  |  |
| Control | 4 | 10 | 0.077 | 12.5 | 1.089 | 143.432 |
| Lipids（+）+FBG（+）+BMI（+） |  |  |  |  |  |  |
| Longevity | 0 | 0 |  |  |  |  |
| Control | 8 | 0 | 0.238 | 0.139 | 0.012 | 1.608 |
|  |  |  |  |  |  |  |
