# Supplementary material for: Identification of cardiovascular health gene variants related to longevity in a Chinese population
Source: Aging (Albany NY). 2020 Sep 7;12(17):16775–802. doi: 10.18632/aging.103396 (PMC7521493; doi:10.18632/aging.103396)
Supplement: Supplementary Table 9 [file aging-12-103396-s008..pdf]

SUPPLEMENTARY TABLE

Supplementary Table 9. Distribution of TFPI rs7586970 genotype in study samples included in meta-analysis.

Year	Country	Sample	N			Total
			TT	TC	CC	
2007[56]	Australia	170 cases and 162 controls	158	145	29	332
2007[57]	Australia	26 cases and 56 controls	59	21	2	82
2008[58]	Denmark	57 cases and 103 controls	86	61	13	160
2009[59]	Japan	175 cases and 1684 controls	1574	276	9	1859
2010[60]	Norway	138 cases and 409 controls	267	280	0	547
2017[61]	China	1271 cases and 1287 controls	2159	146	253	2558
